# COVID-19 employment shocks and safety net expansion: Health effects on displaced workers^☆^

**DOI:** 10.1016/j.ssresearch.2024.103059

**Published:** 2024-11

**Authors:** Daniel Schneider, Kristen Harknett, Annette Gailliot

**Affiliations:** a Harvard Kennedy School, USA; b University of California, San Francisco, USA; c University of California, Berkeley, USA

## Abstract

COVID-19 precipitated sharp job losses, concentrated in the service sector. Prior research suggests that such shocks would negatively affect health and wellbeing. However, the nature of the pandemic crisis was distinct in ways that may have mitigated any such negative effects, and historic expansions in unemployment insurance (UI) may have buffered workers from negative health consequences. We draw on employer-employee linked cross-sectional (N = 15,219) and panel (N = 3307) data from service sector workers to estimate the effects of job loss on health and wellbeing during COVID-19. Using employer fixed-effects, lagged dependent variables, and models that focus on job loss due to establishment closure to minimize confounding, we find negative effects of unemployment on health and wellbeing. However, in periods when UI was most generous or in cases where UI fully replaced pre-job loss wages, unemployed workers who received UI were no worse off than those who remained employed. Although UI protected against worsening health, receiving generous UI benefits did not confer a health advantage relative to working at the height of the pandemic.

## 1 Introduction

The COVID-19 crisis represented the single largest shock to employment since WWII. In the early weeks of the outbreak, the unemployment rate surged from less than 4% in February to almost 15% in April of 2020. These job losses were most heavily concentrated in retail, food service, and hospitality, a set of industries that we refer to as “the service sector.” Prior research has documented robust relationships between job loss and mental and physical health outcomes (Burgard et al. 2007; Brand 2015). The COVID-19 pandemic then had the potential to deliver twinned health shocks via direct disease transmission and via adverse health consequences of unemployment.

Yet, the nature of job loss during the COVID-19 pandemic was distinctive in the United States relative to past economic crises in ways that may have significantly diminished the negative effects of job loss on health and wellbeing. First, unlike in previous recessions, hourly service sector workers were most affected, but most research that finds negative effects of job loss on health focuses on blue-collar or professional workers (Brand 2008; Strully 2009), who arguably have “more to lose” than precariously-employed service sector workers (see Brand 2015; Suppa 2021 for reviews). Second, pandemic-related job losses were caused by a public health crisis that precipitated widespread unemployment, which could have reduced the negative consequences of the stigma of job loss (e.g. Clark 2003). Third, the counter-factual condition to job loss, remaining on the job, materially changed given the occupational hazards posed by in-person service sector work during COVID-19 (Wolfe et al., 2021), potentially changing the relative costs of job loss.

Additionally, the crisis of pandemic-related job loss was met by an uncharacteristically generous expansion of unemployment insurance (UI) through the Coronavirus Aid, Relief, and Economic Security (CARES) Act, which may have reduced the negative consequences of job loss. However, while the CARES Act substantially increased the reach and generosity of unemployment insurance, the effectiveness of the administration of the program varied widely across states and the expansion was time-limited (Pandemic Oversight Committee 2023), leading to plausibly exogenous variation in access to these benefits.

We take up the questions of how COVID-19 related job losses affected the health and wellbeing of workers in the service sector compared to those who remained on the job and how effectively the social safety net response buffered these workers from adverse health effects of job loss. We draw on unique data collected from a cross-section of 15,219 employed and recently unemployed (i.e. laid off or furloughed) service sector workers surveyed by the Shift Project between April and October of 2020 along with longitudinal data that followed an occupational cohort of 3307 service sector workers from 2019 through 2020. These data contain detailed measures of the reasons for job loss, the UI claims process, and worker wellbeing. Respondents were currently or recently employed at one of 136 of the largest retail or food service firms in the United States, which together employed 10.5 million workers in 2019, or approximately 60% of employment in those sectors nationally (Authors’ calculations from the 2019 Reference USA data file and the 2019 American Community Survey file). Focusing on this large and impacted sector also provides an opportunity to make comparisons among a relatively homogeneous sample that varies, though, in their recent experience with job loss and UI receipt.

We make two primary contributions to knowledge on job loss, wellbeing, and the safety net. First, we estimate the consequences of job loss for the health and wellbeing of service sector workers during the pandemic. We build on prior literature on the effects of job loss on health and wellbeing that has directly addressed the empirical challenge that job loss is not random and may be selected on confounding characteristics or even the result, rather than solely the cause, of diminished health and wellbeing. Research on job loss has been attentive to these threats to validity, taking advantage of exogenous shocks to employment during recessions or due to plant closures to address selection (Brand et al., 2008; Kessler et al., 1987). We deploy these tools to examine whether job loss still produces negative effects on health and wellbeing even during the unique conditions of the COVID-19 pandemic.

Second, existing research also leaves important questions unanswered about how effectively the safety net response buffers workers from the negative health effects of job loss. European research has leveraged variation in social safety net generosity to address this important question. However, research on this question in the European context contends with problems of confounding in the cross-national comparisons and further, this work does not generally adopt the methods for causal identification of the effect of job loss when examining the moderating role of safety-net generosity (e.g. Ouweneel 2002; Tøge 2016). Research in the U.S. context is much more limited, with area level studies contending with problems of ecological fallacy (Cylus et al., 2014; Wu and Evangelist 2022) and very little work at the individual level that attends to variation in patterns of UI receipt, selection into unemployment, and health. We assess if these historic expansions in unemployment insurance during COVID, which nevertheless were still uneven and often inaccessible, buffered the effects of job loss on health and wellbeing.

### 1.1 Job loss and health and wellbeing

Labor market earnings are the primary source of income for most working-aged adults, and, consequently, job loss typically leads to a sudden and sharp loss of economic resources and declines in the standard of living (Couch et al., 2011). A loss of economic resources can have negative and disruptive effects on health behaviors such as diet, exercise, and sleep, on preventive and primary health care, and the management of chronic disease conditions (Monsivais 2015; Voβemer et al., 2018). The loss of economic resources is a major pathway through which job loss, especially when accompanied by long periods of unemployment, is expected to exact a toll on physical and mental health (Burgard and Kalousova 2015).

Beyond being a primary source of income, a rich sociological literature has shown that work is a source of identity and meaning, social belonging, and structure (Newman 1988; Elder, 1998). A loss of a job, therefore, also has disruptive effects that go beyond the loss of earnings. Job loss represents not just the loss of a paycheck but also a major social dislocation, including a severing of usual daily and weekly routines and of workplace relationships. Losing one's job can also trigger a crisis of confidence, self-blame, anxiety and depression (Newman 1988; Chen, 2015; Rao, 2020).

Given the centrality of labor market participation for economic sustenance, social inclusion, identity, and the structuring of life, it is no surprise that a voluminous literature has found that job loss has a host of negative effects on health (i.e. Brand 2015). A central finding of this literature is that job loss has widespread downstream effects on workers’ health and well-being, including their physical health and longevity, their health-related risk behaviors, and their mental health and psychological well-being. The negative effects of job loss on psychological health include increased psychological distress and unhappiness (Brand et al., 2008; Kessler et al., 1987) as well as changing sleep patterns (Blanchflower and Bryson 2021) and worsening self-rated health (Burgard et al., 2007; Schaller and Stevens 2014).

This well-documented, expected, and robust connection between job loss and health belies underlying methodological and conceptual complexities. Methodologically, establishing a causal connection between job loss and health is complicated by the possibility of reverse causality and confounding. For the former, those who experience physical or mental health problems may be more likely to leave or lose their jobs for health reasons and to be in worse health, not because of job loss, but because of a pre-existing health issue (Kessler et al. 1987). For the latter, unobserved characteristics that cause job loss may also cause diminished health and wellbeing, leading to spurious estimates of the effects of job loss on these outcomes (Stevens, 2018).

The literature on job loss and health and wellbeing has been quite attentive to these problems of inference (Kessler et al., 1987; Strully 2009; Stevens, 2018), focusing on strategic sites for inquiry that provide a kind of causality by design. One such approach is to focus on periods of economic recession based on the logic that job loss in such times is more likely to be exogenous with respect to individual worker characteristics (Brand 2015; Stevens, 2018). However, recession studies have not reached a clear consensus about how economic downturns affect health, because the findings differ when the recessionary shock is measured at the aggregate level with mortality as the ultimate health outcome, or measured at the individual-level with the focus on physical or mental health outcomes (Burgard and Ailshire, 2013; Burgard and Kalousova 2015).

Conceptually, the relationship between job loss and health is complicated by the circumstances surrounding the job loss. In particular, job loss that is experienced as a large-scale and collective shock is expected to have different effects on displaced workers than individual job loss (Newman 1988). Research has examined whether the negative consequences of job loss might be diminished when layoffs are more prevalent, such as during broad recessions, making layoffs seem less driven by individual failures (Aquino et al., 2022; Brand et al., 2008; Clark 2003). While this idea is plausible, a long line of research suggests that unemployment in the United States continues to carry negative effects even during widespread recessions due to stigma and loss of self-worth and social status (Elder 1974; Komarovsky 1940; Newman 1988), and comparative cross-national work finds the individual effects of unemployment may be worse when unemployment levels are high (Calvo et al. 2015).

### 1.2 Job loss and health and wellbeing during COVID-19

Addressing the question of how COVID-19 related job losses affected the health and wellbeing of displaced workers is complicated by the same methodological and conceptual challenges described above, but also by the unprecedented nature of the COVID-19 shock.

In the early portion of the pandemic in the Spring of 2020, job loss was rapid and widespread due to government mandated business shutdowns to slow the spread of the COVID-19 virus. The COVID-19 pandemic and recession were especially severe for workers in the service sector. Sharp curtailments in customer demand and local safety mandates led employers to lay off their workers, precipitating a 15% reduction in service sector employment from pre-pandemic levels (Bureau of Labor Statistics, 2022) . The unusual rapidity and severity of the job losses in the early months of the pandemic, coupled with the fact that this labor market shock came without warning, meant that these job losses were largely exogenous. Further, the nature of the employment shock meant that job loss was likely to be seen as a collective rather than an individual phenomenon, thus normalizing or destigmatizing unemployment for displaced workers.

The disproportionate job losses in the service sector are another distinctive feature of the COVID-era labor market shock. Prior research on the causal effects of job loss on health and wellbeing has often focused on blue-collar workers (Browning and Heinesen 2012; Chen, 2015; Kuhn et al., 2009; Schiele and Schmitz 2016). It is unclear how generalizable these results are to service sector workers, who often report low wellbeing even while employed compared to those in other sectors (Schneider and Harknett 2019). One study, by Dooley et al. (2000) finds that, controlling for poverty, employment in a “bad job” is equivalent to unemployment in terms of negative effects on depression. More broadly, the negative consequences of unemployment appear to be diminished for workers with prior exposure to economic insecurity (Burgard et al., 2007; Maroto 2015), lending further credence to the idea that job losses in the service sector, which befell a group of highly precarious workers in high-turnover jobs (Choper et al., 2021; Schneider and Harknett 2021), might not carry negative consequences. In contrast, Witteveen and Velthorst (2020) found that European workers with lower occupational prestige (such as domestic cleaners) experienced more negative effects of job loss than those with higher occupational prestige (such as managing directors) during COVID-19.

Research on the effects of job loss is inherently a comparative question. Because we cannot observe displaced workers in the counterfactual state of remaining employed, workers who remain employed serve as a natural approximation of that counterfactual. In the context of the COVID-19 pandemic, this counterfactual comparison is complicated by the fact that working conditions underwent profound change, especially for front-line workers in the service sector (Wolfe et al., 2021). During the COVID-19 pandemic, these workers faced high risks of COVID-19 transmission (Phuong et al. 2021) and suffered disproportionate mortality (Chen et al., 2021) even as they dealt with what appears to have been an increasingly fractious and confrontational public. Despite these hazards, on-the-job protections remained very limited, as expanded paid sick leave in the Families First Coronavirus Response Act was circumscribed to those at mid-sized firms (Jelliffe et al., 2021), and voluntary employer supports were piecemeal and time limited (Ho et al., 2020; Kinder et al., 2022).

As in previous periods, job loss during COVID-19 could have had negative effects on workers’ health and wellbeing. But, the health effects of job loss during COVID-19 could have also been muted by the aforementioned particular circumstances of the pandemic. A growing literature has taken up this empirical question and begun to examine the consequences of job loss during COVID-19 for health and wellbeing (Mai et al., 2023).

One noteworthy study by Schieman et al. (2023) drew on a fortuitously timed longitudinal survey, which included Canadian workers who lost jobs in the early months of the pandemic. In stark contrast to the vast literature documenting the harmful effects of job loss for displaced workers, these authors found that job loss was associated with a *reduction* in psychological distress relative to remaining employed in the first few weeks of the pandemic. By May of 2020, the benefit of a layoff (relative to remaining employed) had subsided. This study complemented the survey findings with 47 in-depth interviews and argued that the pandemic job losses were seen by some as a “forced vacation” and as an opportunity for leisure, rest, or family time. Key to this positive account of job loss was the expectation that it was temporary, with the majority of workers expecting to soon return to their jobs. Reinforcing this finding on temporary job loss, studies in the United Kingdom and Sweden found that while permanent job losses had negative effects on mental health outcomes such as depression and anxiety, these mental health effects were absent when the job losses were temporary furloughs (Wang et al., 2022; Blomqvist et al., 2023).

In the U.S. context, where government and employer welfare supports following job loss are weaker than in Canada or Europe, the relationship between job loss and wellbeing may be different. Much less work takes up this issue in the United States. Two studies do so by drawing on city-specific samples of working parents. Kalil et al. (2020) drew on a sample of 572 parents in Chicago and found that job loss during COVID-19, when concurrent with income loss, was negatively related to physical, psychological, and familial wellbeing. In a similar survey, Gassman-Pines et al. (2020) studied 561 hourly service workers in Philadelphia with a young child and found that parent and child mental health worsened in the short-term as the families faced income and job loss. Both studies provided valuable early insights into COVID-19 and job loss but were limited to small samples of parents in a single city, which, given geographic differences in COVID-19 response and safety net efficacy, limits generalizability. Further, both studies covered quite brief time frames, with Kalil et al. (2020) examining a period of two months and Gassman-Pines et al. (2020) relying on 30 days of daily text surveys.

Several other studies examine somewhat broader populations and find that COVID-era job losses were negatively associated with health outcomes. A Colorado study using data from February to June of 2021 found that unemployed workers reported worse physical and mental health than the employed and that this pattern persisted even upon reemployment (Hanel 2022). Extending to the national level, Umucu and coauthors (2022) studied Mechanical Turk data from May to June of 2020 and found that stress, anxiety, and loneliness were more likely to be reported by those who experienced job loss. In related work focusing on job insecurity rather than job loss and unemployment, Donnelly et al. (2022) used the Household Pulse Survey from April 2020 to March 2021 and found increased depression and anxiety related to perceived job insecurity. In another study, using a national survey of 2,000 respondents fielded by Qualtrics from July to October of 2020, both COVID-related job loss and furloughs were associated with higher levels of depression and anger compared to employed workers (Grace 2023).

In sum, the evidence on COVID-era job losses in the U.S. echoes the familiar finding that job loss is negatively related to health, but the emerging literature on job loss during COVID has not yet deployed the tools for causal inference that have been developed and applied in the pre-COVID era. These emerging studies have also not incorporated the potential moderating effects of the unemployment insurance safety net, the topic to which we now turn.

### 1.3 Does unemployment insurance protect the health of displaced workers?

Any negative effects of job loss may also be reduced when workers who have lost their jobs can access unemployment insurance or other safety net programs that provide economic support. These programs are likely to buffer workers against the negative consequences of job loss to the extent that the negative effects of job loss on health and wellbeing flow via an economic resource pathway.

There is substantial indirect evidence that this economic pathway is likely to play an important role in generating adverse health consequences of job loss. First, upon job loss, workers often face reduced or complete income reduction. This income loss is severe in the short term and heightened during economic recessions (Brand 2015; Voydanoff 1984). Workers report earnings losses even if unemployment is only a temporary displacement (Brand 2003; Cha and Morgan 2010). Second, economic losses caused by job loss in turn negatively affect health and wellbeing. Research has examined this question by estimating the residual effect of job loss on wellbeing after controlling for income loss. Financial insecurity plays an important role in explaining diminished health and wellbeing upon job loss in Britain (i.e. Clark 2003; Hald Andersen, 2009), Germany (i.e. Gerlach and Stephan 1996; Knabe and Rätzel 2007), and Sweden (Korpi 1997). Finally, governmental programs such as UI, can effectively allow the unemployed to cover their living expenses and prevent large drops in consumption (East and Kuka 2015). Conversely, the exhaustion of UI benefits is associated with significant declines in family income, increases in poverty (Rothstein and Valletta 2017), and reductions in consumer spending (Ganong and Noel 2019).

However, there is little existing research that directly examines the degree to which access to unemployment insurance benefits effectively buffers those who have lost jobs from adverse health consequences. One line of European research finds mixed results, with some evidence that more generous social spending moderates the negative effects of unemployment (Korpi 1997; Nordenmark et al., 2006; Tøge 2016), but other evidence finds no association between overall generosity of social welfare benefits payments and the wellbeing of the unemployed (Ouweneel 2002). However, these papers either contend with the limitations of single-country case studies in particularly generous contexts (Sweden) or with the problems of confounding at the country level in cross-national studies. These papers also use a simple bivariate measure of unemployment and do not attempt to identify any causal effects of unemployment.

In the U.S., a series of studies have deployed data at the state level to estimate if the association between state-level health outcomes and unemployment rates is moderated by state-level UI generosity, finding evidence of such moderation for opioid overdose deaths (Wu and Evangelist 2022) and suicide (Cylus et al., 2014). However, these aggregate estimates do not take up the question of selection into unemployment and cannot discern if the improved health outcomes associated with more generous UI accrue to the unemployed or more generally in the population (Sjöberg 2010).

Much less work takes up this crucial question at the individual level. A small number of studies link state-level UI eligibility or generosity with individual-level health outcomes to find partial moderation of the effect of unemployment on health outcomes by UI (Kuka 2020; Young 2012). However, rather than observe UI receipt directly, each of these studies relies on state UI benefit rules. These approaches essentially provide “intent-to-treat” estimates. However, given wide cross-state variation in UI recipiency rates, even after conditioning on likely eligibility for UI benefits, (Forsythe and Yang 2021), these intent-to-treat estimates may not provide accurate estimates of the “treatment-on-the-treated” effects of actually receiving UI payments.

### 1.4 Did the COVID-era unemployment insurance expansions protect the health of displaced workers?

The role of UI in buffering any adverse consequences of job loss during COVID-19 is especially salient since the Federal government responded to the rapid shift in employment levels during COVID-19 by significantly increasing the generosity of unemployment insurance, notably passing the Coronavirus Aid, Relief, and Economic Stimulus (CARES) Act. The CARES Act temporarily expanded both benefit amounts and eligibility. By some estimates, augmented UI led to replacement rates in excess of 100% of earnings for large shares of low-wage workers (Ganong et al. 2020).

However, the CARES Act provisions also added complexity to a UI system that was already complicated and widely variable across states. Prior to CARES, states already had varying UI rules, with states such as Florida and North Carolina offering a maximum of 12 weeks of benefits while the state average was 25 weeks (CRS, 2019). In the years prior to the pandemic, workers faced significant hurdles in the process of applying for UI (Badger and Parlapiano 2020). Workers needed to document their job searches weekly, experienced long response times, and technical glitches on state websites made it difficult for workers to receive and stay qualified for UI benefits.

These existing inefficiencies were exacerbated by the unprecedented levels of initial UI claims filed in the wake of COVID-19 (Zipperer and Gould 2020). While the Pandemic Unemployment Assistance (PUA) program under the CARES Act broadened UI eligibility criteria, allowing some with low or irregular earnings to newly qualify, many workers did not know of the program or had trouble applying (Kovalski and Scheiner 2020). PUA rollout timing also varied widely between states, with some states requiring applicants to first go through the regular application process and be rejected before applying for the PUA program. This was exacerbated by technical difficulties with online systems and overloaded call centers (Gould-Werth 2020). As states developed UI infrastructure in response to COVID-19, highly publicized UI fraud led states to enact measures that ultimately further slowed UI receipt for many eligible individuals (Donnan and Pickert 2021). These administrative burdens create significant variation in actual UI receipt. By November 2020, only three states, North Dakota, Rhode Island, and Wyoming, were able to meet federal guidelines of getting benefits out to 87% of applicants within three weeks (Pew 2020).

A set of important studies examines how the broader state context of social safety net generosity buffered the mental health consequences of income shocks and economic stress during COVID-19, finding an important buffering effect (Donnelly and Farina 2021; Farina et al., 2023). However, few studies take up the important specific question of how the CARES Act UI provisions buffered the negative consequences of unemployment for households. Most of this work focuses on the effects of UI receipt on household economic security. Work in this vein finds that unemployed workers who received UI in the spring of 2020 experienced less material hardship (Karpman and Acs 2020), that UI lessened food insecurity (Raifman and et al., 2021), that spending declined among the unemployed following FPUC expiration (Farrell et al., 2020), and that financial fragility increased (Schneider and Tufano, 2020). In terms of health and wellbeing, both Berkowitz and Basu (2021) and Carey, et al 2021 use the Household Pulse Survey data and find that, compared to unemployed workers who received UI, unemployed workers without UI benefits experienced more food insufficiency, financial instability, health care delays, and increased depression and anxiety symptoms.

### 1.5 Our approach

We draw on unique data collected by The Shift Project over the course of the COVID-19 pandemic, which includes repeated cross-sections and longitudinal data collected from large samples of employed and not employed service sector workers and detailed measures of reasons for job loss, unemployment insurance receipt, and household economic security and worker wellbeing.

First, we use these data to provide estimates of the effects of job loss on service sector workers during the first months of COVID-19. We do so in series of models that combine the strengths of prior recession and plant closure designs. By focusing on a period of mass layoffs, we reduce the risk of selection into job loss. Advancing existing research, we are also able to estimate firm-fixed effects models to make within-firm comparisons of currently employed workers with those recently laid-off from the same firms. Additionally, as detailed in supplementary materials, we also are able to use fine-grained measures of the reasons for job loss to contrast job loss stemming from store closures against job loss that was individually selective, and we are able to leverage longitudinal data to estimate effects of job loss on well-being outcomes, controlling for health selection. These data allow us to provide credible estimates of the effect of job loss during COVID-19 for a key population of workers.

Second, we leverage detailed measurement of UI application and receipt to estimate the degree to which the safety net response protected those who lost their jobs from adverse health consequences. The Shift Project data contain direct and detailed measures of UI receipt during the COVID-19 pandemic as well as measures of economic insecurity and hardship. The CARES Act provided for the possibility of generous UI payments that had the potential to completely replace lost income. However, state-to-state differences in the practical accessibility of UI benefits and the expiration of benefits led to significant variation in actual UI receipt. We leverage this variation in UI receipt and deploy fine-grained measurement of exposure to the UI process to estimate the degree to which UI receipt reduced the health consequences of job loss, and we leverage change over time in UI generosity to estimate the degree to which variation in the amount of UI shaped the extent to which UI receipt mitigated adverse health consequences.

## 2 Data and methods

### 2.1 The Shift Project data

We draw on novel survey data collected by The Shift Project from 10,684 respondents who were currently or recently employed in the service sector (retail, pharmacy, grocery, hardware, electronics, general merchandise, fast food, casual dining, delivery and fulfillment, hotel) surveyed between April and June and an additional sample of 4,535 hourly workers surveyed between September and October 2020. Collectively, these samples include respondents at 136 of the largest service sector firms in the United States. These COVID-19-era cross-sections are part of the larger Shift Project survey, which began surveying hourly workers in the service sector in 2017.

To collect this data, The Shift Project utilizes a novel sampling and recruitment design in which a sample of workers employed at large, named retail and food service establishments were recruited using targeted advertisements on Facebook and Instagram. The Shift Project first creates employer-specific “audiences” of Facebook and Instagram users using Meta's targeted advertising platform. This platform allows advertisers to construct “audiences” of users with specific characteristics, including education, place, age, and, crucial for the Shift Project's purposes, employer. For each of the 136 targeted firms, firm specific audiences were constructed by entering accepted spellings of the employer as well as variants in order to construct as comprehensive an audience as possible. While Facebook is opaque about how exactly employer is identified for users, it appears to be both a function of explicit listing of employer on users' profiles and a prediction-based approach. However, because this implied employer characteristic is not immediately updated, The Shift Project was able to recruit respondents who were employed at particular firms as well as their counterparts who had recently experienced separation from these same firms.

**Appendix Fig. B3:**
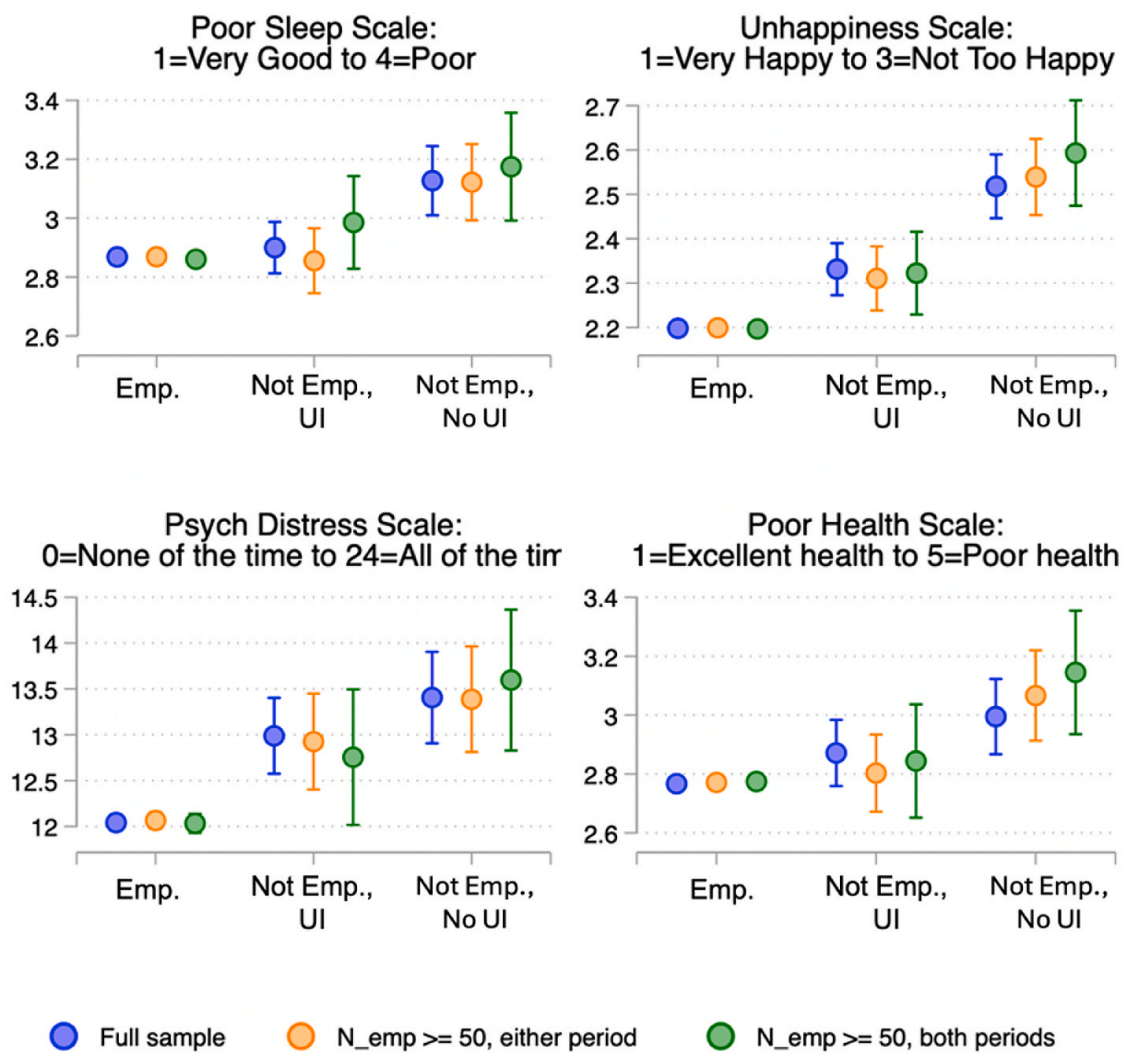
Predicted Values of Health Outcomes by Employment Status, UI Receipt, and Employer Size Note: This figure corresponds to Fig. 4 and is a robustness check on sample size by employer.

These firm-specific audiences then serve as a quasi-sampling frame. At each wave of data collection, the Shift Project team selects a set of companies, within the project budget constraint, to target for survey recruitment. The selected set includes a core of 30 firms that are targeted at each wave as well as additional firms selected for repeated coverage across waves and for balance across sub-sectors. For the selected set of companies, The Shift Project then constructed unique advertisements corresponding to each of these audiences. For each firm, the advertisement included a photograph of a worker in a setting designed to resemble their workplace and an employer-specific recruitment message (e.g., “Current or former Walmart worker? Please take our survey”). For each of the two waves of data collection used in the analysis sample, the set of targeted firms was divided into groups of 8–10 firms and each of those sets of advertisements was run for six days (from 12AM on Friday through 12AM on Thursday). The result is that there is partial, but not complete, overlap of the firms at which respondents are/were employed across the two waves. In Appendix Figure B3, we show the robustness of our results to (1) restricting the sample to only include respondents who were currently/recently employed at firms with at least 50 respondents per wave or (2) restricting the sample to only include respondents who were currently/recently employed at firms with at least 50 respondents per wave in both waves. The results are not sensitive to these restrictions, though the standard errors are slightly larger, reflecting the diminished sample size.

Respondents who saw and clicked on the advertisement were then taken to an online survey hosted on the Qualtrics platform. They were asked to consent to participation and then directed to a set of screening questions. First, respondents were asked for their employment status with options of (1) “I am employed,” (2) “I was furloughed by my employer (I am not getting any scheduled hours),” (3) “I was recently laid off and am now unemployed,” and (4) “none of the above.” In round 9, respondents were also presented with two additional options: (5) “I quit my job and am now unemployed” and (6) “I am retired (no longer working). Respondents who selected option (4) (“none of the above”) were skipped out of the survey. Respondents who selected option (1) (“I am employed”) were asked the name of their employer, with closed-ended responses for their targeted employer (e.g., an advertisement delivered to a Walmart audience included an option for Walmart as the employer) as well as a set of other closed-end options listing similar employers (e.g. Target, Costco, Sam's Club) and an “other” open-text entry. Workers who selected options (2), (3), (5), or (6) were presented with a similar question, asking about their former employer. We limit our analysis sample to workers in groups (1) employed, (2) furloughed, or (3) laid off.

Pooling across the data collected in these advertisements, respondents were then assigned to a (former) employer based on their selection of a closed-ended response or on their write-in response. Write-in responses were manually inspected and cleaned. Respondents who wrote-in a firm other than one in the Shift Project target population were excluded from the analysis (<1% of respondents). The sample was further limited to hourly workers, but not restricted by occupation and managers paid on an hourly basis were included in the sample. The analysis sample then contains both respondents at the firms targeted in a given survey round as well as other respondents who were delivered advertisements, took the survey, and were current/recent workers at a firm in the Shift Project target population, but whose current/recent employer was not directly targeted. The survey asked respondents to report on their job conditions if employed, their demographics, economic circumstances, receipt of UI, and health, among other topics.

Our sample is a non-probability sample, and we are attentive to the potential for bias. These biases could stem from both non-coverage of our target population and selectivity of respondents on observed and unobserved attributes. With respect to our quasi sampling frame, we estimate from Pew Survey data that eighty percent of all working Americans use Facebook or Instagram and engagement is high with 80% of users reporting daily use of the platforms (author's calculation from 2018 Pew Survey of Social Media Use).

We expect that the larger potential selection bias is that of selection into actually taking the survey. Prior work estimates that 1.2% of respondents who viewed the recruitment advertisement progressed and at least partially completed the survey (Schneider and Harknett 2022). We address selection bias on demographic characteristics including age, gender, and race/ethnicity by constructing and applying post-stratification weights that align the characteristics of our survey sample with those of service sector workers in the American Community Survey. We post-stratify and weight the data to the demographic characteristics – age, gender, and race/ethnicity - of workers surveyed in the ACS who were employed in the same set of industries and occupations. These industry and occupations are described in Appendix Table A2. We contrast the demographics of our unweighted and weighted sample against those of the American Community Survey (ACS) benchmark in Appendix Table A3.

The sample may nevertheless be selective on unobserved attributes, which would not be addressed by this weighting approach. In earlier methodological work, Schneider and Harknett (2022) devise a novel test for selection into the Shift Project survey on an unobserved confounder. They specify likely confounders of the association between job quality and wellbeing on which response could be biased, run recruitment advertisements that make such confounders salient (e.g. “Hate your job at Walmart?” or “Love your job at Walmart”) and then assess if key associations differ between those recruited through the channels. They find no evidence of effect modification.

Additional reassurance that the Shift Project sample more generally accurately reflects the broader population of workers is provided by Schneider and Harknett (2022) in comparing the Shift data with gold standard probability samples (the Current Population Survey and the National Longitudinal Survey of Youth), subsampled to align with the Shift data on occupation and industry. Their analyses find that the Shift survey data yields estimates of wages, tenure, and the wage/tenure relationship that are closer to each of the two probability samples than the probability samples are to one another. These and additional checks are reported in Schneider and Harknett (2022).

While the Shift data have some important limitations, the data also have unique strengths. First, the data focuses on workers in a large and policy-relevant sector of the economy. While the data cannot be used to generalize to the total labor force, the sample composition imposes some internal validity by design, limiting analytic comparisons to workers in similar circumstances. Second, the data provide much more detailed measures of job quality than found in standard labor force surveys, alongside reports of household economic security and worker wellbeing. Third, the data also provide rare employer-employee linked data, with large samples of workers nested within the same identifiable firms, which allows for the estimation of employer fixed-effects models. We leveraged these unique strengths of the Shift Project data to investigate the effects of the COVID-19 crisis on service sector workers.

#### 2.1.1 Key variables

*Employment Status*. We construct a simple dichotomous measure of employment status in the pooled cross-section. Respondents were asked “What is your employment status?,” and we code respondents as either being employed or not employed.

*Unemployment Insurance.* In both the Spring 2020 and Fall 2020 cross-sections, we asked respondents detailed questions about UI receipt. All workers who were currently not employed from being laid-off, furloughed, or quitting their job were asked if they applied for UI in 2020. We then asked follow-up questions and were able to identify where they were in the application process at the time of survey: 1) Did not attempt to apply, 2) Completed an application but hadn't received a reply or payments, 3) Completed an application and was denied, 4) Completed an application, was approved, but had not yet received benefits, or 5) Applied and received benefits. Comparing those who had applied and received benefits (#5) to those who had applied and had not yet heard back (#2), we construct a UI-receipt variable. This comparison allows us to impose significant homogeneity in the comparison, excluding respondents who had not applied or were denied and instead relying on differences in UI administration response-speed to identify the effects of UI receipt. While this removes much heterogeneity, there may still be some due to the fact that some respondents who had not yet heard back may in the end be denied benefits.

We also asked respondents who had received UI directly about how their UI benefits compared to their prior earnings. Respondents were asked “How does that [how much received in unemployment insurance] compare to what you were earning from your job before you began receiving unemployment insurance?”, with response options of “Much more than I was making”, “More than I was making”, “About the same as I was making”, “Less than I was making”, and “Much less than I was making.”

*Health and Wellbeing.* We gauged adult health and well-being with four measures. First, we used a psychological distress scale that includes the six items from the Kessler-6 index of non-specific psychological distress (list items as validated in Prochaska et al., 2012). The scale of psychological distress that combines these six items has a Cronbach's α reliability of 0.93. Second, we measure self-rated sleep quality as very good, good, fair, or poor; this follows the 4-point PSQI Likert scale validated in Muzni et al. (2021). Third, we gauge happiness by asking respondents, “taken all together, how would you say things are these days? Would you say you are, (1) very happy, (2) pretty happy, or (3) not too happy (following the style of the General Social Survey). Fourth, we model self-rated health, reported as excellent, very good, good, fair or poor (validated in Schnittker and Bacak 2014). In supplemental results, we show that our results are robust to instead operationalizing these four variables dichotomously (Appendix B).

*Control Variables.* The relationship between unemployment and wellbeing could be confounded by various socioeconomic characteristics. We adjust for these by including controls for gender, race/ethnicity, age, marital status, whether a language other than English is spoken at home, school enrollment, educational attainment, and the presence of children in the household. In some cases, we control for month of survey, and we also introduce two measures to control for COVID-19 exposure: whether the respondent has contracted COVID-19 and/or whether any of the respondent's immediate family members have contracted COVID-19. The survey also collects a report of annual household income. However, because we are focused on a period of rapid change in employment and income, we do not use this measure as it is unlikely to reflect these dynamics (Pew 2020).

#### 2.1.2 Analytical approach

Our analysis proceeds in two parts. We first estimate a series of models to identify the effect of job loss on the health and wellbeing of service sector workers during COVID-19. We then investigate the degree to which UI receipt buffered workers from adverse health consequences of job loss, investigating heterogeneity in this protective effect by UI generosity.

*Effects of Job Loss on Health and Wellbeing.* We first draw on the cross-sectional data pooled across Spring and Fall 2020 to estimate OLS models of the relationship between job loss and our measures of health and wellbeing. We begin with the full set of worker controls, then introduce industry fixed-effects, and then introduce employer fixed-effects. This third set of models provides the within-employer effect of unemployment, contrasting workers who remain employed at a given firm and those who recently separated from the same firm. While valuable for reducing unobserved heterogeneity, such models are very rarely, if ever, estimated in the literature, especially on an analytic sample that is composed of multiple firms, due to the lack of employer-employee linked U.S. data. We then focus on the Fall 2020 data, which allow us to include a control for prior COVID-19 infection.

In Appendix C, we describe and present two additional analytic approaches designed to further isolate the effect of job loss on worker health and wellbeing: (1) leveraging exogenous shocks to job loss from whole-store closure and (2) leveraging panel data that allows for the inclusion of lagged dependent variables.

*Buffering Effects of Unemployment Insurance.* In the second part of our analysis, we examine the extent to which UI receipt buffered not employed respondents from the negative effects of being not employed on health and wellbeing. This analysis also proceeds in several steps.

**Appendix Fig. D1:**
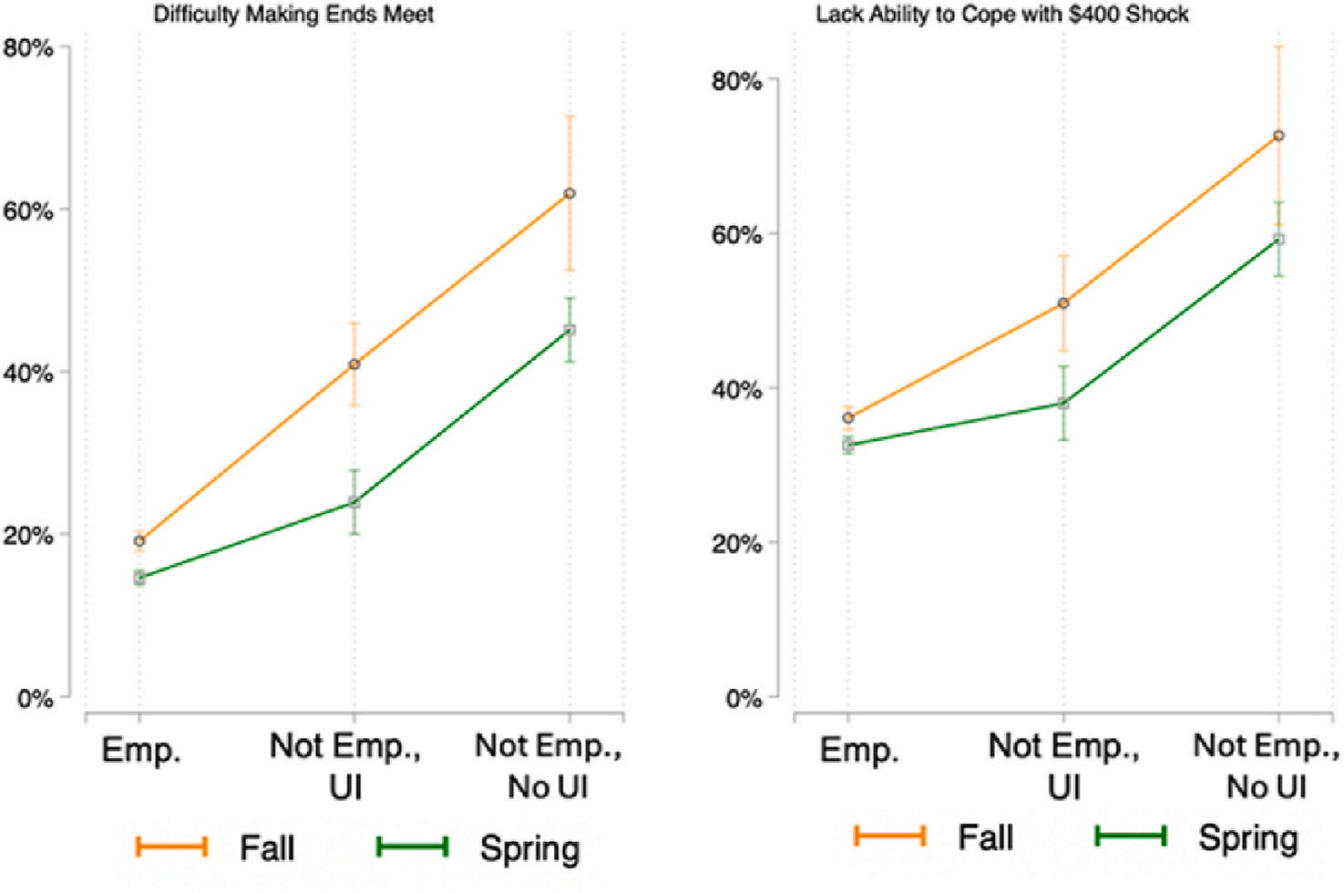
Predicted Values of Association between Unemployment Insurance Benefits and Economic Hardship/Wellbeing by Time Period, Cont.

**Appendix Fig. D2:**
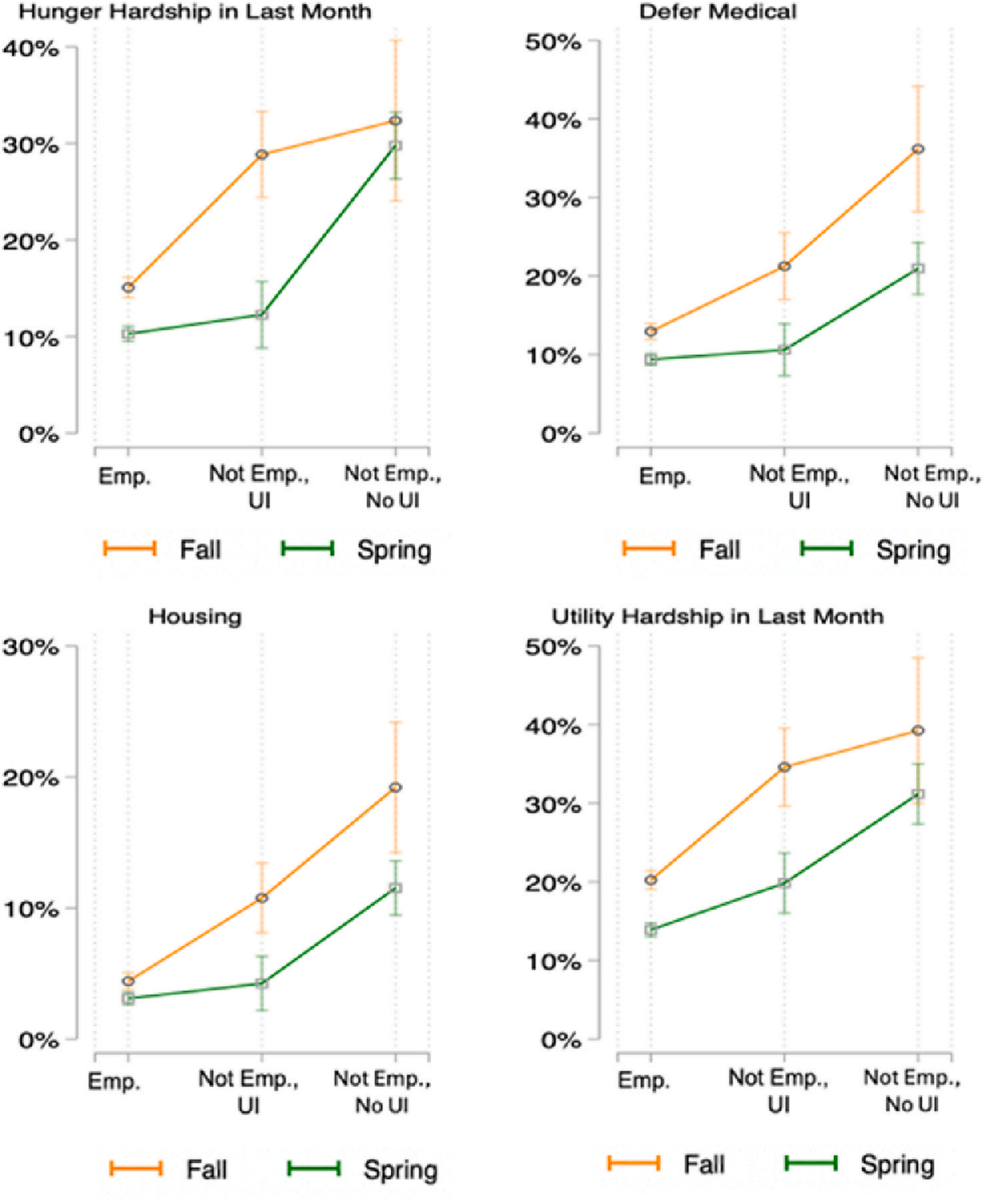
Predicted Values of Association between Unemployment Insurance Benefits and Economic Hardship/Wellbeing by Time Period.

First, we leverage the fact that UI receipt was far from universal and varied significantly across people and state systems. We pool respondents across Spring and Fall 2020 and contrast respondents who (1) remained employed with those who (2) experienced job loss but received UI and with those who (3) experienced job loss, had applied for UI, but had not yet heard back on their applications. Of particular importance, we omit respondents who had not applied for UI or who had been denied UI in order to reduce unobserved heterogeneity. We estimate OLS models that take each of our four measures of health and wellbeing as the outcome, the three-category employment status/UI receipt indicator as the primary independent variable, and then control for the full set of worker characteristics as well as employer, state, and month fixed-effects. Under typical conditions, we would expect job loss to be associated with worse health relative to remaining employed; however, the stressful and risky working conditions during the pandemic could have negated or even reversed this expected relationship. Among those who are not employed, our expectation is that those receiving UI would fare better than those who applied UI and were awaiting a response. These differences are hypothesized to run through an economic channel and in Appendix Figures D1 and D2, we present results from the same set of models as above, but taking measures of economic security as the outcome variables.

Second, we estimate if the degree to which UI receipt buffered respondents against adverse health and wellbeing consequences varied by UI generosity. We do so with two separate analyses – one that exploits variation over time in UI generosity and one that exploits variation in UI generosity relative to pre-job-loss wages.

**Fig. 1:**
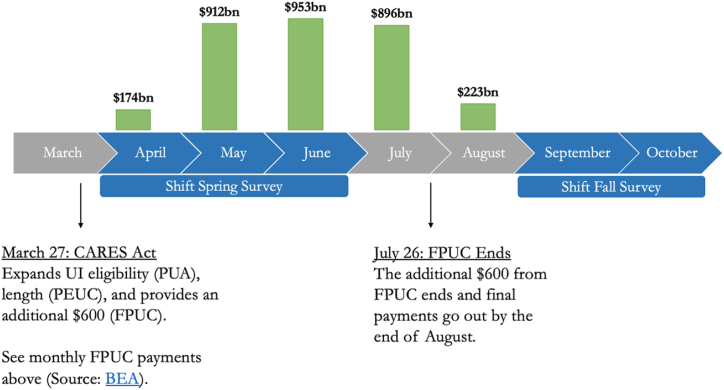
Timeline of FPUC Payments in 2020 Notes: PUA (Pandemic Unemployment Assistance) expanded eligibility for people who were not typically eligible for unemployment insurance, e.g. gig workers and independent contractors. PEUC (Pandemic Emergency Unemployment Compensation) extended the number of weeks an individual could receive unemployment benefits, up to 53 weeks. FPUC (Federal Pandemic Unemployment Compensation) provided an additional $600 in weekly benefits. These three programs adapted unemployment insurance during COVID-19 in different ways – through broadening eligibility (PUA), extending duration (PEUC), and increasing payment amounts (FPUC).

For the first, we exploit the fact that UI generosity varied substantially over time between April of 2020 and October of 2020, the period that we capture in our survey. The months April–June of 2020 correspond to the months when the Federal Pandemic Unemployment Compensation (FPUC) was most generous, providing a $600 supplement, and months September to October of 2020 correspond to the months when the FPUC had expired. The timeline of these provisions according to the U.S. Bureau of Economic Analysis is shown in Fig. 1.

We leverage this variation in generosity by timing to examine if *when* respondents received UI is predictive of the extent to which they were buffered against the health effects of job loss. We do so by estimating a model that interacts our measure of UI receipt (employed, not employed and without UI benefits, not employed and receiving UI benefits) with survey round (Spring, 2020 or Fall, 2020) to predict health and wellbeing. If generosity shapes the degree to which UI receipt buffers respondents against the adverse effects of job loss, we would expect to see larger associations between UI receipt and health and wellbeing in the Spring of 2020 than in the Fall of 2020.

For the second, we pool the data from Spring and Fall (2020) and examine UI generosity directly at the individual level. We compare respondents who reported that (a) they remained employed, (b/c/d) were not employed and received UI that was [more/the same/less] than they were making when last employed, or (e) were not employed and did not receive UI. We estimate this model with our standard set of controls and employer fixed-effects. If the negative effects of job loss operate only through the economic pathway, then we would expect that respondents whose UI benefits equaled or exceeded prior income would not be negatively affected by job loss and that therefore their health outcomes would be no worse than their employed counterparts.

## 3 Results

### 3.1 Unemployment and wellbeing

Table 1 shows clear evidence that job loss is negatively associated with worker health and wellbeing, with consistently significant estimates across four indicators of wellbeing. In M1, we show that workers who were not employed at interview, as compared with those who remained employed, had significantly worse sleep (b = 0.13, p < .001), lower happiness (b = 0.21, p < .001), more psychological distress (b = 1.20, p < .001), and worse self-rated health (b = 0.10, p < .05). The largest effects are for happiness, at about one-third of a standard deviation, and there are somewhat smaller effects for psychological distress at about one-quarter of a standard deviation.

**Table 1:** Association between employment status and health outcomes (continuous outcomes).

	Sleep Quality						Happiness					
	1 = Very Good to 4 = Poor						1 = Very Happy to 3 = Not Too Happy					
	M1	M2	M3	M4	M5	M6	M1	M2	M3	M4	M5	M6
Not Employed	0.13***	0.14***	0.13***	0.15**	0.12	0.11	0.21***	0.22***	0.21***	0.24***	0.17**	0.15**
(Mean)	(2.88)	(2.88)	(2.88)	(2.88)	(2.87)	(2.87)	(2.22)	(2.22)	(2.22)	(2.22)	(2.20)	(2.20)
Demographic Controls	✔	✔	✔	✔	✔	✔	✔	✔	✔	✔	✔	✔
State Fixed-Effects						✔						✔
Industry Fixed-Effects		✔						✔				
Employer Fixed-Effects			✔	✔	✔	✔			✔	✔	✔	✔
COVID-19 Exposure						✔						✔
Month Fixed-Effects						✔						✔
Pooled Data	✔	✔	✔				✔	✔	✔			
Round 8 (Spring, 2020) Data Only				✔						✔		
Round 9 (Fall, 2020) Data Only					✔	✔					✔	✔
Observations	15,219	15,219	15,219	10,684	4535	4535	15,219	15,219	15,219	10,684	4535	4535

	Psychological Distress						Self-Rated Health					
	0 = None of the time to 20 = All of the time						1 = Excellent health to 5 = Poor health					
	M1	M2	M3	M4	M5	M6	M1	M2	M3	M4	M5	M6
Not Employed	1.20***	1.13***	1.13***	0.94***	1.56**	1.50***	0.10*	0.09*	0.16*	0.13*	0.29**	0.30**
(Mean)	(12.13)	(12.13)	(12.13)	(12.08)	(12.26)	(12.26)	(2.78)	(2.78)	(2.78)	(2.80)	(2.72)	(2.72)
Demographic Controls	✔	✔	✔	✔	✔	✔	✔	✔	✔	✔	✔	✔
State Fixed-Effects						✔						✔
Industry Fixed-Effects		✔						✔				
Employer Fixed-Effects			✔	✔	✔	✔			✔	✔	✔	✔
COVID-19 Exposure						✔						✔
Month Fixed-Effects						✔						✔
Pooled Data	✔	✔	✔				✔	✔	✔			
Round 8 (Spring, 2020) Data Only				✔						✔		
Round 9 (Fall, 2020) Data Only					✔	✔					✔	✔
Observations	15,219	15,219	15,219	10,684	4535	4535	15,219	15,219	15,219	10,684	4535	4535

Note: M1 – M3 analyze pooled data including Spring 2020 and Fall 2020. M4 analyzes only Spring 2020 data. M5 and M6 use only Fall 2020 data. ***p < .001, **p < .01, *p < .05.

In Model 2 we introduce industry fixed-effects alongside the individual-level controls. The estimates are essentially unchanged. In M3 of Table 1, we leverage the employer-employee linked data and estimate a set of employer fixed-effects. These models restrict the comparisons between employed and not employed workers to those at the same firms – e.g., currently employed McDonald's workers compared with recently laid off or fired McDonald's workers. Compared with prior work on plant closures and on unemployment during recessions, this approach gives us very tightly defined comparison groups. Strikingly, we again find similarly sized coefficients on job loss predicting sleep, happiness, psychological distress, and self-rated health. In short, among similarly situated workers, one recently laid off or fired from a firm and the other still employed at the same firm, we find negative and significant associations between job loss and wellbeing.

These estimates begin to establish the case for a causal effect of job loss on health and wellbeing during the pandemic. One possible threat to causal inference is that contracting COVID-19, or having a family member who contracted COVID-19, could precipitate both job loss and negative effects on health and wellbeing. COVID-19 exposure was not measured in Spring (2020) but was measured in the Fall. In M4 and M5 of Table 1, we first separately estimate the four key models by Fall versus Spring of 2020. The estimates are similar in each round for sleep and happiness, but somewhat larger in Fall of 2020 for psychological distress and self-rated health. Then, in M6, focusing on Fall of 2020, we show that these associations are quite robust to controlling for COVID-19 exposure. All of these estimates are quite consistent when we operationalize the dependent variables dichotomously (Appendix Table B1).

In Appendix C, we report the results of two supplementary analyses designed to further isolate the causal effects of job loss on health and wellbeing, namely, leveraging an exogenous shock to employment due to the closure of an entire store and leveraging panel data that allows us to include lagged dependent variables. As described in Appendix C, the results are consistent across these alternative approaches.

In sum, focusing on a period of large-scale and rapid mass layoff and furlough, we find evidence of significant negative effects of job loss on sleep quality, happiness, psychological-distress, and self-rated health. These effects are robust to controls, to within-industry comparisons, and even to employer fixed-effects, and they do not appear to be driven by COVID-19 exposure.

#### 3.1.1 Buffering Effects of Unemployment Insurance on the negative consequences of unemployment

The COVID-19 crisis was unique not just for the speed and severity of the economic downturn, but for the generosity of the social safety net policy response. We exploit variation in the accessibility of this generous support to assess the effectiveness of the safety net response in buffering those who lost their jobs both economically and from negative health shocks.

**Fig. 2:**
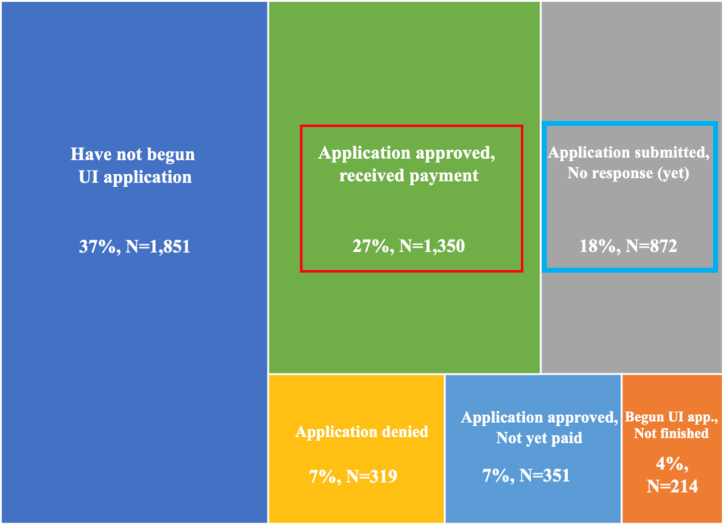
Percentage of workers in each stage of the UI process Note: Overall sample size is 4956.

Source: Shift Project surveys

**Fig. 3:**
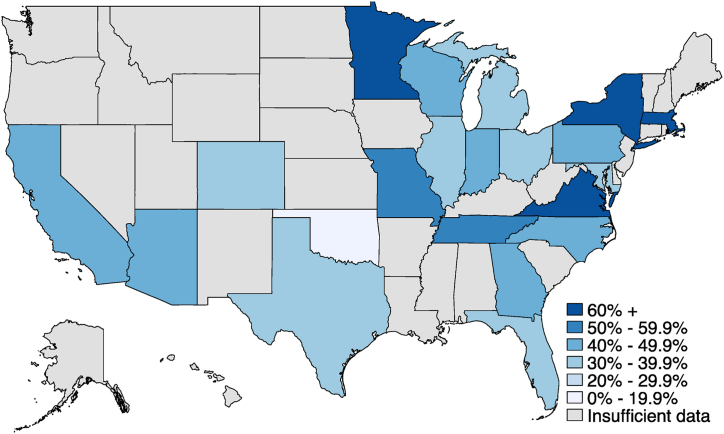
Share of UI applicants reporting having received benefits, by state.

Source: Shift Project surveys

A key source of variation in unemployment insurance benefits is the simple difference between receiving the benefits and not receiving the benefits. In the Spring and Fall 2020 surveys, we included detailed questions designed to gauge workers’ progress through the UI system as of the date of interview. As shown in Fig. 2, we find substantial funneling of workers through the system, with just 27% of workers who had been laid-off or furloughed reporting receipt of UI benefits by the time of interview (Fig. 2, red box). It is possible that workers who did not apply or who were denied benefits might be negatively selected. The construction of these questions allows us to create a tightly defined comparison, contrasting workers who received UI with workers who had applied, but not yet heard back on their applications (Fig. 2, blue box), and excluding those who did not apply or who were denied from the comparison. Difficulty accessing UI also played out very differently across states. As shown in Fig. 3, the share of workers who applied and received benefits by the time of survey varied dramatically, from 78% in Minnesota to 17% in Oklahoma.

In comparing those who received UI with their counterparts who applied and were awaiting a reply, we are primarily interested in the extent to which receiving UI buffered workers against the negative effects of job loss on health and wellbeing. However, the primary channel for such effects is economic. We would expect that for UI to buffer workers against negative health and wellbeing effects of job loss, UI would need to reduce economic insecurity. In Appendix D, we show that was indeed the case. Workers who received UI, especially when UI levels were most generous, were much less economically disadvantaged relative to workers who remained employed than were workers who did not receive UI.

We next assess the effectiveness of UI receipt in protecting workers who lost jobs from adverse consequences for health and wellbeing by comparing the health and wellbeing of workers in 2020 who remained employed, with those who lost their jobs and received UI payments, and with those who lost their jobs and had applied for UI but had not yet heard back. Including the comparison with stably employed workers at the same firms from which displaced workers were laid off or furloughed addresses the question of whether job loss during the pandemic might have been protective relative to going to work in-person during the risky, uncertain, and stressful pandemic period.

**Fig. 4:**
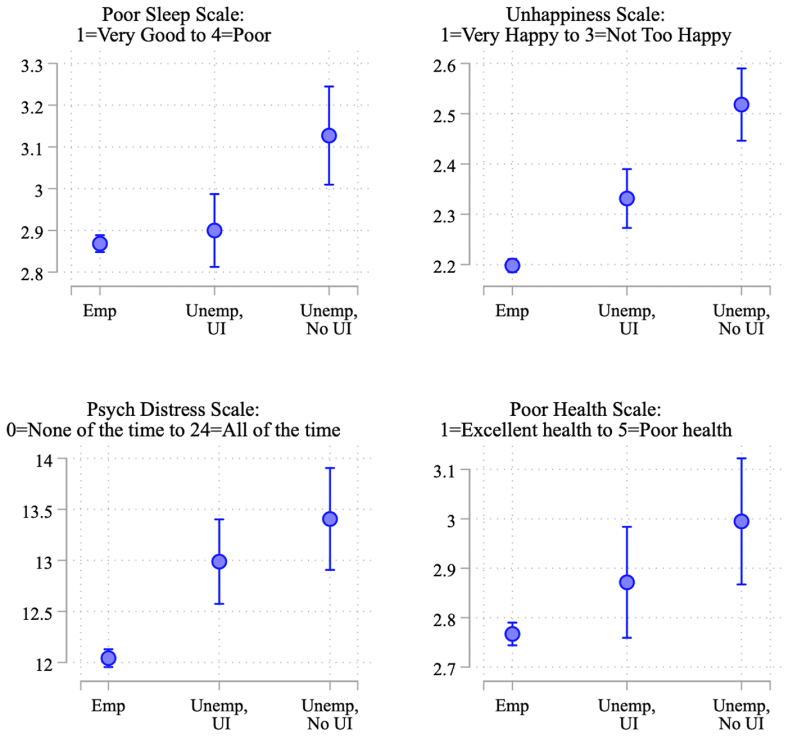
Predicted values of health outcomes by employment status and UI receipt.

Fig. 4 plots predicted levels of health and wellbeing for these three groups of workers, derived from the model estimates presented in Table 2. Employed respondents consistently fare best, as seen in their relatively low predicted values for poor sleep quality, unhappiness, psychological distress, and poor health. In contrast, not employed respondents who had applied for UI but had not heard back fared significantly worse than those who remained employed (differences between employed and not employed without UI benefits are statistically significant at p < .001 in each model). These effect sizes are also substantive, ranging from a fifth of a standard deviation (for self-rated health) to half a standard deviation (for happiness).

**Table 2:** Association between employment status and unemployment insurance receipt and health outcomes in 2020.

	M1	M2	M3	M4
	Poor Sleep Scale	Happiness Scale	Psychological Distress Scale	Poor Health Scale
	1 = Very Good to 4 = Poor	1 = Very Happy to 3 = Not Too Happy	0 = None of the time to 24 = All of the time	1 = Excellent Health to 5 = Poor Health
Employed	−0.03	−0.13***	−0.95***	−0.10
Not Employed, UI	(ref)	(ref)	(ref)	(ref)
Not Employed, no UI	0.23**	0.19***	0.42	0.12
Demographic Controls	✔	✔	✔	✔
Employer Fixed-Effects	✔	✔	✔	✔
Pooled Data	✔	✔	✔	✔
(Mean)	(2.88)	(2.22)	(12.13)	(2.78)
N	15,219	15,219	15,219	15,219

Note: All models in Table 2 analyze pooled data from Spring (2020) and Fall 2020. ***p < .001, **p < .01, *p < .05.

In contrast, respondents who had lost their jobs but had already received UI by the time of interview were generally significantly less adversely impacted by job loss than those who were waiting for a response to their UI application. In the case of sleep, there was no significant or substantive difference between respondents who remained employed and those who had lost their jobs but received UI (b = −0.032; p = .50), even as those who had lost their jobs and not yet received UI fared significantly worse than those who lost jobs and had received UI (b = 0.227, p < .001). In terms of happiness, UI receipt partially buffered the negative effects of job loss. Respondents who lost their jobs but had received UI fared significantly worse than those who remained employed (p < .001) but significantly better than those who lost jobs but had not yet received UI (p < .001). This same gradient, indicative of partial buffering, is evident in Fig. 4 for poor self-rated health. However, for psychological distress, it appears that UI receipt fully buffered the negative consequences of job loss. These model estimates provide no evidence that those who received UI during the pandemic fared better than their counterparts who remained employed and worked during the first year of the pandemic.

These models pool together those who received UI in Spring of 2020 and in Fall of 2020 However, there was significant variation in UI benefits amounts over this time period. While UI was significantly augmented during the pandemic, this increase in generosity was short-lived, binding from March to July, but expired by August, as shown in Fig. 1. We next assess the difference that this benefits reduction made in the ability of UI to buffer workers from the adverse effects of job loss.

**Fig. 5:**
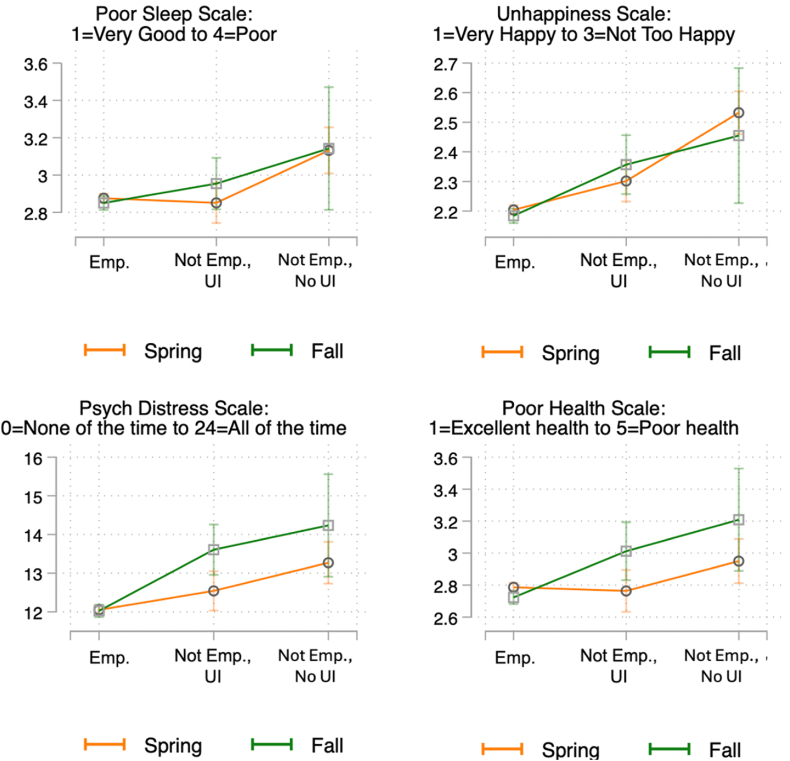
Predicted values of health outcomes by employment status and Unemployment Insurance Receipt, Spring 2020 versus fall 2020.

A first way to examine the importance of UI generosity (rather than simple receipt) is to pool the data across Spring and Fall 2020 and interact our three-category measure of employment/UI receipt with whether the respondent was interviewed in Fall of 2020, when the $600 supplement was no longer in effect. We plot predicted values from these models (regression results are shown in Appendix Table B2) in Fig. 5. We find that not employed workers who did not receive UI reported significantly worse health outcomes in both Spring and Fall of 2020 compared to employed workers. However, for workers who had lost their jobs and had already received UI, this support appears to have more effectively protected workers against adverse health and wellbeing consequences in the Spring of 2020, when UI was most generous, than in the Fall of 2020, when UI had been scaled-back. For sleep quality, psychological distress, and poor health, we see that the slope of the orange line, plotting predicted values in the Spring of 2020, is essentially flat between employed workers and those who had lost their jobs but received UI, while workers in Spring (2020) who had lost jobs but not received UI fared significantly worse than their counterparts. In contrast, the slope on the green line, capturing predicted values of the outcomes in Fall (2020), when UI benefits had been scaled back, is consistently positive across groups, showing that UI remained protective, but incompletely so in the context of reduced benefits.

A second way to examine the importance of UI generosity is to directly examine how UI benefits generosity affected wellbeing. If respondents whose UI payments equaled or exceed prior earnings fared as well as the employed, but respondents whose UI payments fell short of prior earnings fared less well, then that would be consistent with the economic channel being the primary effect pathway. Table 3 shows exactly this pattern. Not employed respondents whose UI payments equaled their prior earnings fare no worse in terms of sleep quality than those who remained employed (and those whose payments exceeded prior earnings actually fare marginally better (b = −0.19, p < .05)), while those whose UI payments were less than prior earnings slept significantly worse than the employed (b = 0.12, p < .01), though not as poorly as those who were not employed but had not received UI (b = 0.29, p < .001). We see the same pattern for happiness, psychological distress, and self-rated health, though for those outcomes, UI payments exceeding prior income is not associated with a statistically significant benefit over remaining employed. Even under the most generous UI benefit conditions in which lost earnings were fully replaced by benefits, those who worked during the pandemic reported similar mental health and self-rated health compared with those who were laid off or furloughed and receiving UI benefits.

**Table 3:** Associations between employment status, UI amount, and health outcomes.

	M1	M2	M3	M4
	Poor Sleep Scale	Happiness Scale	Psychological Distress Scale	Poor Health Scale
	1 = Very Good to 4 = Poor	1 = Very Happy to 3 = Not Too Happy	0 = None of the time to 24 = All of the time	1 = Excellent Health to 5 = Poor Health
Employed	(ref)	(ref)	(ref)	(ref)
UI: More than I was making	−0.19*	0.02	0.20	−0.11
UI: Same as I was making	0.06	−0.03	0.71	0.12
UI: Less than I was making	0.12**	0.14***	1.06***	0.09
Not Employed, No UI	0.29***	0.30***	1.54***	0.19***
Demographic Controls	✔	✔	✔	✔
State Fixed-Effects	✔	✔	✔	✔
Employer Fixed-Effects	✔	✔	✔	✔
Month-Fixed Effects	✔	✔	✔	✔
Pooled Data	✔	✔	✔	✔
(Mean)	(2.88)	(2.22)	(12.13)	(2.78)
N	15,219	15,219	15,219	15,219

Note: All models in Table 3 analyze pooled data from Spring (2020) and Fall 2020. ***p < .001, **p < .01, *p < .05.

## 4 Discussion

As the COVID-19 pandemic surged, so too did job losses, especially for low-wage workers. The large U.S. service sector, which employs nearly 1 in 5 U.S. workers across retail, food service, and hospitality sectors, was particularly hard hit. While prior research suggests that such widespread job losses would be likely to have negative effects on workers’ health and wellbeing, the distinct circumstances of the pandemic, including widespread unemployment, focused effects on already vulnerable workers, deteriorating conditions even for those who remain employed, and the unprecedented safety net response, make the overall effects of job loss on health and wellbeing for service sector workers during COVID-19 an open question.

Drawing on Shift Project data from a national sample of over 15,000 workers currently employed or recently displaced from retail, food service, and hospitality jobs and longitudinal data from over 3,000 workers, we find robust and consistent evidence of negative effects of job loss on workers' happiness, sleep quality, psychological distress, and self-rated health. Research on job loss and health faces challenges in sorting out health effects of job loss from selection or reverse causality, because those who are displaced from their jobs often differ in a number of relevant ways from their employed counterparts. In our analyses, we are able to combine many of the strengths and avoid some of the weaknesses of prior research. First, we employ a strong comparison group, by comparing currently employed workers with workers from the exact same firms who had recently experienced job loss, as is done in research on layoffs. But, importantly, we do so at a time when displaced workers are far less selective than usual, because the exogenous shock of the pandemic led to a sudden and drastic rise in unemployment. Second, in the tradition of prior research distinguishing reasons for job loss (Strully 2009), we compare workers who lost their jobs due to establishment closure with those who were laid off. Here, unlike prior research, we are able to align the occupational backgrounds of workers displaced by layoffs with those displaced by establishment closures. In each case, workers were formerly employed at large retail or food service establishments. Third, we address health selection by using panel data to test the robustness of our results to a set of lagged dependent variable model specifications. Each of these approaches yields consistent evidence that job loss during the COVID-19 pandemic had a harmful effect on displaced workers’ physical and mental health.

Our study reinforces and corroborates the findings from a set of studies that have found negative associations between pandemic-era job losses and health outcomes in a variety of local and some national data sources (Kalil et al., 2020; Gassman-Pines et al., 2020; Hanel 2022; Umucu et al., 2022; Donnelly et al., 2022; Grace 2023). However, in contrast to this set of studies, ours deploys the tools of causal inference developed in the pre-pandemic period to minimize the possibility that reverse causality or omitted variables drive associations between job loss and health (Brand 2015).

The harmful effects of job loss for worker health are striking in the context of pandemic conditions that made going to work at a service sector job a physical health risk. Service sector workers frequently interact with customers and often work in heavily trafficked, dense workplace settings in which they are unable to maintain social distance, putting them at heightened and continual risk of COVID exposure. In this context, unlike during non-pandemic times, we might expect job loss to have a protective effect on measures of well-being. In fact, Schieman et al. (2023) present evidence that, early in the pandemic, displaced Canadian workers viewed job loss as a “forced vacation,” and experienced lower levels of psychological distress than their employed counterparts. However, these benefits had already dissipated by May of 2020. In our results for the U.S. service sector, negative effects of job loss on psychological distress, and also sleep, happiness, and self-rated health are very much evident during the first year of the pandemic, even for displaced workers who would have been likely to face health risks and stressful conditions at work had they remained employed. Further, these negative health effects are also striking considering the overall low-quality job conditions faced by those employed in service sector jobs in the U.S. These results speak to the centrality and salience of labor force participation, not just as a source of earned income, but also as a source of identity and meaning, and of structure for workers’ everyday life.

The COVID-19 pandemic was distinctive not just in the rapidity and severity of job loss but also in the unusually generous policy response. In particular, unemployment insurance replacement rates far exceeded their usual levels, and more displaced workers were eligible for these benefits by virtue of new expansions. We contribute to a small literature on the effects of UI in buffering the adverse health consequences of job loss (e.g. Kuka 2020; Young 2012; Berkowitz and Basu 2021; Carey, et al 2021; Farina et al., 2023). We find that in the first year of the pandemic, UI partially buffered workers from the negative effects of job loss on sleep quality, happiness, psychological distress, and self-rated health. Further, in the earliest months of the pandemic, when the economic effects of job loss were more fully buffered for workers displaced from low-wage work, so too are the health effects of job loss. Although this pattern of results provides evidence that UI benefits do have a protective effect, other differences between the Spring of 2020 and the Fall of 2020 periods could also contribute to differences in health outcomes. However, we find further evidence that UI benefits were operative when we directly measure workers’ reported UI wage replacement levels. Those workers for whom lost earnings were fully replaced by UI benefits reported similar health outcomes compared with their counterparts who kept their jobs.

Data from the Shift Project were uniquely suited to address the research questions at hand but have some important limitations that should be kept in mind. Foremost, the data are from a non-probability sample of workers. Although the data were weighted to reflect the demographic attributes and educational attainment levels of service sector workers in the broader population and have been validated against probability samples (Schneider and Harknett 2022), the sample may nevertheless differ from the broader population of workers on unobserved attributes. If our sample differs systematically from the general population on attributes that are potential effect modifiers of the relationship between job loss and health, the estimates we present could be biased. While we find no evidence of this bias when comparing the Shift data to probability data sources, we cannot entirely rule out this potential source of bias. Also, our sample does not capture workers without internet access or those who speak a language other than English. Our sample is also limited to workers currently or formerly employed at large retail, food service, or hospitality firms and does not capture workers employed at smaller establishments. The results we present, therefore, have some limits to their generalizability.

The COVID-19 pandemic represents the worst health shock and one of the most severe economic contractions of the last century. Drawing on timely and rich data from the Shift Project, our paper provides robust evidence that displaced workers experienced declines in their physical and mental health during the pandemic. Importantly, we also find that during this time of enormous challenge and hardship, unemployment insurance offered not just economic sustenance but also protected against deteriorating physical health and mental health. Those who received unemployment insurance that fully replaced their lost wages were able to sustain a steady level of sleep quality and self-rated health and experienced muted negative effects on happiness and psychological distress compared with their counterparts who were not able to access unemployment insurance. However, the UI expansions of early 2020 have not been sustained, and these benefits are no longer as generous or accessible as they were in the early pandemic period. Our research shows that this retrenchment of the UI safety net poses a real threat to the well-being of current and future displaced workers.

## CRediT authorship contribution statement

**Daniel Schneider:** Writing – review & editing, Writing – original draft, Supervision, Resources, Methodology, Investigation, Funding acquisition, Formal analysis, Conceptualization. **Kristen Harknett:** Writing – review & editing, Writing – original draft, Supervision, Resources, Methodology, Investigation, Funding acquisition, Conceptualization. **Annette Gailliot:** Writing – review & editing, Writing – original draft, Investigation, Formal analysis.

## Figures and Tables

**Appendix Table A1:** Employer Sample Sizes

Employer	Spring 2020	Fall 2020	Panel Baseline 2019	Employer	Spring 2020	Fall 2020	Panel Baseline 2019
7-Eleven	17	2	2	Express	.	63	.
Ace Hardware	68	90	37	Family Dollar	.	.	40
Advance Auto Parts	8	82	2	Fedex	144	140	20
Albertsons	125	95	6	Five Guys	7	.	.
Aldi	106	67	77	Food Lion	226	98	.
Amazon	217	99	123	Fred Meyer	.	.	93
American Eagle	1	.	.	GameStop	69	63	78
Apple	32	.	3	Gap	129	80	19
Applebee's	244	70	2	Giant	48	62	.
Arby's	179	94	4	Giant Eagle	65	65	.
AT&T	.	.	4	Hannaford	62	72	27
AutoZone	1	1	.	Harbor Freight Tools	.	.	1
Banana Republic	.	.	16	Hardee's	8	55	.
Barnes & Noble	4	81	18	Harris Teeter	30	16	1
Bath & Body Works	.	.	1	HEB	55	117	.
Bed Bath & Beyond	.	87	2	Hilton	57	.	17
Best Buy	71	122	93	Hobby Lobby	118	146	.
Best Western	50	1	7	Holiday Inn	61	1	17
Bob Evans	49	60	.	Home Depot	310	112	118
Buffalo Wild Wings	48	55	4	HomeGoods	.	.	17
Burger King	191	89	42	Hy-Vee	141	94	.
Carl's Jr.	.	1	.	Hyatt	41	.	2
Cheesecake Factory	87	28	5	IHOP	81	78	3
Chick-Fil-A	127	96	101	Ikea	101	43	24
Chili's	52	73	.	In–N-Out Burgers	61	31	37
Chipotle	83	84	76	Jack in the Box	1	1	4
Costco	197	103	65	Jason's Deli	13	.	.
Cracker Barrel	126	91	122	JCPenney	91	152	55
Culvers	18	.	.	Jiffy Lube	33	.	.
CVS	232	131	104	Jimmy John's	62	.	37
Dairy Queen	95	1	2	KFC	34	.	31
Days Inn	2	.	4	Kmart	.	82	13
Denny's	23	49	.	Kohls	4	156	73
DHL	4	28	5	Kroger/QFC	306	112	92
Dick's Sporting Goods	.	64	3	La Quinta	1	.	5
Dillard's	1	.	.	Little Caesars	1	.	.
Disney	1	75	.	Lowe's	293	115	141
Dollar General	275	140	53	Macy's	100	94	81
Dollar Tree	106	116	86	Marriott	119	2	38
Domino's	128	83	76	Marshalls	10	83	35
Dunkin Donuts	115	108	54	McDonald's	292	89	108

Employer	Spring 2020	Fall 2020	Panel Baseline 2019	Employer	Spring 2020	Fall 2020	Panel Baseline 2019
Meijer	122	88	50	Verizon	.	.	3
Michaels	1	104	73	Victoria's Secret	1	.	83
Nordstrom	1	.	14	Waffle House	200	82	.
O'Reilly Auto Parts	75	64	34	Walgreens	341	127	113
Office Depot	53	38	2	Walmart	430	180	97
Old Navy	.	.	105	Wawa	48	.	51
Olive Garden	215	108	57	Wegmans	76	.	59
Outback Steakhouse	20	.	1	Wendy's	92	120	69
P.F. Chang's	31	.	.	Whataburrger	6	54	.
Panda Express	60	.	1	Whole Foods	145	87	76
Panera	92	107	143	Wyndham	52	.	11
Papa John's	154	71	2	XPO Logistics	28	.	6
Petco	99	104	76	Zaxby's	5	.	.
PetSmart	142	116	69	N	11,641	8015	4813
Pizza Hut	186	107	62				
Publix	357	152	58				
QuikTrip	67	62	21				
Rally's	1	.	.				
Red Lobster	41	87	1				
Red Robin	66	2	4				
Rite Aid	100	108	6				
Ross	2	76	46				
Safeway	207	121	96				
Sams Club	118	85	65				
Sears	31	57	13				
Shaw's	29	55	.				
Shell	22	.	.				
ShopRite	66	86	7				
Smith's Food and Drug	25	3	.				
Sonic	85	69	31				
Staples	74	1	3				
Starbucks	308	128	250				
Stop & Shop	43	116	40				
Subway	286	108	83				
T.J. Maxx	.	.	48				
Taco Bell	138	110	53				
Target	337	140	149				
Texas Roadhouse	10	1	.				
Trader Joe's	56	53	25				
Ulta Beauty	.	68	2				
UPS	240	182	129				

**Appendix Table A2:** ACS Industry and Occupations in Sample

Industry	Title
580	Lumber and building material retailing
581	Hardware stores
591	Department stores
600	Miscellaneous general merchandise stores
601	Grocery stores
620	Auto and home supply stores
621	Gasoline service stations
623	Apparel and accessory stores, except shoes
631	Furniture and home furnishings stores
633	Radio, TV, and computer stores
641	Eating and drinking places
642	Drug stores
651	Sporting goods, bicycles, and hobby stores
682	Miscellaneous retail stores
691	Retail trade, n.s.

**Table:** 

Code	Title	Code	Title
203	Clinical laboratory technologies and technicians	328	Human resources clerks, except payroll and timekeeping
204	Dental hygienists	329	Library assistants
205	Health record tech specialists	335	File clerks
206	Radiologic tech specialists	336	Records clerks
207	Licensed practical nurses	337	Bookkeepers and accounting and auditing clerks
208	Health technologists and technicians, n.e.c.	338	Payroll and timekeeping clerks
214	Engineering technicians, n.e.c.	344	Billing clerks and related financial records processing
217	Drafters	347	Office machine operators, n.e.c.
218	Surveyors, cartographers, mapping scientists and technicians	348	Telephone operators
223	Biological technicians	349	Other telecom operators
224	Chemical technicians	356	Mail clerks, outside of post office
225	Other science technicians	357	Messengers
226	Airplane pilots and navigators	359	Dispatchers
227	Air traffic controllers	364	Shipping and receiving clerks
228	Broadcast equipment operators	365	Stock and inventory clerks
229	Computer software developers	366	Meter readers
233	Programmers of numerically controlled machine tools	368	Weighers, measurers, and checkers
234	Legal assistants, paralegals, legal support, etc.	373	Material recording, scheduling, production, planning, and expediting clerks
253	Insurance sales occupations	375	Insurance adjusters, examiners, and investigators
254	Real estate sales occupations	376	Customer service reps, investigators and adjusters, except insurance
255	Financial services sales occupations	377	Eligibility clerks for government programs; social welfare
256	Advertising and related sales jobs	378	Bill and account collectors
258	Sales engineers	379	General office clerks
274	Salespersons, n.e.c.	383	Bank tellers
275	Retail sales clerks	384	Proofreaders
276	Cashiers	385	Data entry keyers
277	Door-to-door sales, street sales, and news vendors	386	Statistical clerks
283	Sales demonstrators/promoters/models	389	Administrative support jobs, n.e.c.
308	Computer and peripheral equipment operators	405	Housekeepers, maids, butlers, stewards, and lodging quarters cleaners
313	Secretaries	417	Fire fighting, prevention, and inspection
315	Typists	418	Police, detectives, and private investigators
316	Interviewers, enumerators, and surveyors	423	Other law enforcement: sheriffs, bailiffs, correctional institution officers
317	Hotel clerks	425	Crossing guards and bridge tenders
318	Transportation ticket and reservation agents	426	Guards, watchmen, doorkeepers
319	Receptionists	427	Protective services, n.e.c.
326	Correspondence and order clerks	434	Bartenders

Code	Title	Code	Title
435	Waiter/waitress	516	Heavy equipment and farm equipment mechanics
436	Cooks, variously defined	518	Industrial machinery repairers
439	Kitchen workers	519	Machinery maintenance occupations
443	Waiter's assistant	523	Repairers of industrial electrical equipment
444	Misc. food prep workers	525	Repairers of data processing equipment
445	Dental assistants	526	Repairers of household appliances and power tools
446	Health aides, except nursing	527	Telecom and line installers and repairers
447	Nursing aides, orderlies, and attendants	533	Repairers of electrical equipment, n.e.c.
453	Janitors	534	Heating, air conditioning, and refrigeration mechanics
454	Elevator operators	535	Precision makers, repairers, and smiths
455	Pest control occupations	536	Locksmiths and safe repairers
456	Supervisors of personal service jobs, n.e.c.	539	Repairers of mechanical controls and valves
457	Barbers	543	Elevator installers and repairers
458	Hairdressers and cosmetologists	544	Millwrights
459	Recreation facility attendants	549	Mechanics and repairers, n.e.c.
461	Guides	563	Masons, tilers, and carpet installers
462	Ushers	567	Carpenters
463	Public transportation attendants and inspectors	573	Drywall installers
464	Baggage porters	575	Electricians
465	Welfare service aides	577	Electric power installers and repairers
468	Child care workers	579	Painters, construction and maintenance
469	Personal service occupations, n.e.c	585	Plumbers, pipe fitters, and steamfitters
473	Farmers (owners and tenants)	588	Concrete and cement workers
479	Farm workers	589	Glaziers
485	Supervisors of agricultural occupations	593	Insulation workers
486	Gardeners and groundskeepers	594	Paving, surfacing, and tamping equipment operators
487	Animal caretakers except on farms	595	Roofers and slaters
488	Graders and sorters of agricultural products	596	Sheet metal duct installers
489	Inspectors of agricultural products	597	Structural metal workers
496	Timber, logging, and forestry workers	599	Construction trades, n.e.c.
498	Fishers, hunters, and kindred	614	Drillers of oil wells
505	Automobile mechanics	615	Explosives workers
507	Bus, truck, and stationary engine mechanics	616	Miners
508	Aircraft mechanics	617	Other mining occupations
509	Small engine repairers	634	Tool and die makers and die setters
514	Auto body repairers	637	Machinists

Code	Title	Code	Title
643	Boilermakers	754	Packers, fillers, and wrappers
649	Engravers	755	Extruding and forming machine operators
657	Cabinetmakers and bench carpenters	756	Mixing and blending machine operatives
658	Furniture and wood finishers	757	Separating, filtering, and clarifying machine operators
666	Dressmakers and seamstresses	759	Painting machine operators
668	Upholsterers	763	Roasting and baking machine operators (food)
669	Shoe repairers	765	Paper folding machine operators
675	Hand molders and shapers, except jewelers	766	Furnace, kiln, and oven operators, apart from food
677	Optical goods workers	769	Slicing and cutting machine operators
678	Dental laboratory and medical appliance technicians	773	Motion picture projectionists
679	Bookbinders	774	Photographic process workers
686	Butchers and meat cutters	779	Machine operators, n.e.c.
687	Bakers	783	Welders and metal cutters
688	Batch food makers	785	Assemblers of electrical equipment
694	Water and sewage treatment plant operators	799	Graders and sorters in manufacturing
695	Power plant operators	804	Truck, delivery, and tractor drivers
696	Plant and system operators, stationary engineers	808	Bus drivers
699	Other plant and system operators	809	Taxi cab drivers and chauffeurs
703	Lathe, milling, and turning machine operatives	813	Parking lot attendants
706	Punching and stamping press operatives	823	Railroad conductors and yardmasters
707	Rollers, roll hands, and finishers of metal	824	Locomotive operators (engineers and firemen)
709	Grinding, abrading, buffing, and polishing workers	829	Ship crews and marine engineers
719	Molders, and casting machine operators	844	Operating engineers of construction equipment
726	Wood lathe, routing, and planing machine operators	848	Crane, derrick, winch, and hoist operators
727	Sawing machine operators and sawyers	853	Excavating and loading machine operators
729	Nail and tacking machine operators (woodworking)	859	Misc. material moving occupations
733	Other woodworking machine operators	865	Helpers, constructions
736	Typesetters and compositors	866	Helpers, surveyors
738	Winding and twisting textile/apparel operatives	869	Construction laborers
739	Knitters, loopers, and toppers textile operatives	874	Production helpers
743	Textile cutting machine operators	875	Garbage and recyclable material collectors
744	Textile sewing machine operators	878	Machine feeders and offbearers
747	Pressing machine operators (clothing)	883	Freight, stock, and materials handlers
748	Laundry workers	885	Garage and service station related occupations
749	Misc. textile machine operators	887	Vehicle washers and equipment cleaners
753	Cementing and gluing machine operators	888	Packers and packagers by hand
		889	Laborers outside construction

**Appendix Table A3:** Shift Sample and American Community Survey (ACS) Descriptives

	Shift Sample	Weighted to ACS_wt1	Weighted to ACS_wt3	ACS Sample
	%	%	%	%
*Age*				
18-19	10%	9%	8%	10%
20-29	26%	34%	29%	38%
30-39	15%	18%	17%	18%
40-49	13%	16%	17%	14%
50-59	19%	14%	16%	12%
60+	16%	9%	12%	8%
*Race/Ethnicity*				
White, non-Hispanic	82%	60%	62%	57%
Black, non-Hispanic	3%	11%	11%	13%
Hispanic	9%	20%	18%	22%
Other race, non-Hispanic	6%	9%	9%	9%
*Partnership Status*				
Married or living with partner	31%	28%	29%	27%
*Child status*				
Has children	52%	45%	50%	40%
*Gender*				
Female	70%	50%	55%	53%
*Educational Attainment*				
Less than high school	4%	4%	4%	18%
High school diploma/GED	34%	33%	35%	36%
Some college	37%	38%	37%	30%
Associate's degree	12%	12%	12%	7%
Bachelor's degree	11%	11%	11%	9%
Master's or advanced degree	2%	2%	2%	2%
*School Enrollment*				
Enrolled in school	19%	21%	19%	25%
*English as a second language*	10%	16%	16%	25%

Note: These descriptives are of the pooled sample, including both Spring and Fall 2020.

**Table:** Appendix Table B1 Association between employment status and health outcomes (Dichotomous outcomes).

	Good or Very Good Sleep Quality						Pretty or Very Happy					
	M1	M2	M3	M4	M5	M6	M1	M2	M3	M4	M5	M6
Not Employed	−0.08**	−0.07**	−0.08**	−0.10**	−0.05	−0.04	−0.15***	−0.15**	−0.15***	−0.17***	−0.13*	−0.12*
(Mean)	(0.33)	(0.33)	(0.33)	(0.32)	(0.33)	(0.33)	(0.69)	(0.69)	(0.69)	(0.69)	(0.71)	(0.71)
Demographic Controls	✔	✔	✔	✔	✔	✔	✔	✔	✔	✔	✔	✔
State Fixed-Effects						✔						✔
Industry Fixed-Effects		✔						✔				
Employer Fixed-Effects			✔	✔	✔	✔			✔	✔	✔	✔
COVID-19 Exposure						✔						✔
Month Fixed-Effects						✔						✔
Pooled Data	✔	✔	✔				✔	✔	✔			
Round 8 (Spring, 2020) Data Only				✔						✔		
Round 9 (Fall, 2020) Data Only					✔	✔					✔	✔
Observations	15,219	15,219	15,219	10,684	4535	4535	15,219	15,219	15,219	10,684	4535	4535

	Psychological Distressed						Very Good or Excellent Self-Rated Health					
	M1	M2	M3	M4	M5	M6	M1	M2	M3	M4	M5	M6
Not Employed	0.11***	0.10***	0.09***	0.08**	0.14**	0.14**	−0.00	−0.00	−0.04	−0.04	−0.07	−0.07
(Mean)	(0.69)	(0.69)	(0.69)	(0.69)	(0.71)	(0.71)	(0.74)	(0.74)	(0.74)	(0.73)	(0.77)	(0.77)
Demographic Controls	✔	✔	✔	✔	✔	✔	✔	✔	✔	✔	✔	✔
State Fixed-Effects						✔						✔
Industry Fixed-Effects		✔						✔				
Employer Fixed-Effects			✔	✔	✔	✔			✔	✔	✔	✔
COVID-19 Exposure						✔						✔
Month Fixed-Effects						✔						✔
Pooled Data	✔	✔	✔				✔	✔	✔			
Round 8 (Spring, 2020) Data Only				✔						✔		
Round 9 (Fall, 2020) Data Only					✔	✔					✔	✔
Observations	15,219	15,219	15,219	10,684	4535	4535	15,219	15,219	15,219	10,684	4535	4535

Note: M1 – M3 analyze pooled data including Spring 2020 and Fall 2020. M4 analyzes only Spring 2020 data. M5 and M6 use only Fall 2020 data. ***p < .001, **p < .01, *p < .05.

**Appendix Table B2:** Associations between employment status and unemployment insurance receipt and health outcomes by time period.

	M1	M2	M3	M4
	Poor Sleep Scale	Happiness Scale	Psychological Distress Scale	Poor Health Scale
	1 = Very Good to 4 = Poor	1 = Very Happy to 3 = Not Too Happy	0 = None of the time to 24 = All of the time	1 = Excellent Health to 5 = Poor Health
Employed	0.02	−0.10**	−0.49	0.02
Not Employed, UI	(ref)	(ref)	(ref)	(ref)
Not Employed, No UI	0.28***	0.23***	0.73*	0.19*
Spring 2020	(ref)	(ref)	(ref)	(ref)
Fall 2020	0.10	0.06	1.07**	0.25*
Employed x Fall 2020	−0.13	−0.08	−1.09**	−0.31**
Not Employed, UI x Fall 2020	(ref)	(ref)	(ref)	(ref)
Not Employed, no UI x Fall 2020	−0.09	−0.13	−0.10	0.01
Demographic Controls	✔	✔	✔	✔
State Fixed-Effects	✔	✔	✔	✔
Employer Fixed-Effects	✔	✔	✔	✔
Month-Fixed Effects	✔	✔	✔	✔
Pooled Data	✔	✔	✔	✔
(Mean)	(2.88)	(2.22)	(12.13)	(2.78)
N	15,219	15,219	15,219	15,219

Note: All models in Appendix Table B2 analyze pooled data from Spring and Fall of 2020. ***p < .001, **p < .01, *p < .05.

**Appendix Table C1:** Associations between employment status and health outcomes by reason laid off or Furloughed

	Poor Sleep Scale		Happiness Scale	
	1 = Very Good to 4 = Poor		1 = Very Happy to 3 = Not Too Happy	
	M1	M2	M1	M2
Employed	(ref)	(ref)	(ref)	(ref)
Furloughed/laid off because store temp./permanently closed	0.15	0.15	0.17*	0.15
Furloughed/laid off for other reason	0.10	0.09	0.17**	0.15**
Demographic Controls	✔	✔	✔	✔
State Fixed-Effects		✔		✔
Employer Fixed-Effects	✔	✔	✔	✔
COVID-19 Exposure		✔		✔
Month Fixed-Effects		✔		✔
Round 9 (Fall, 2020) Data Only	✔	✔	✔	✔
(Mean)	(2.87)	(2.87)	(2.20)	(2.20)
N	4535	4535	4535	4535

	Psychological Distress Scale		Poor Health Scale	
	0 = None of the time to 24 = All of the time		1 = Excellent Health to 5 = Poor Health	
	M1	M2	M1	M2
Employed	(ref)	(ref)	(ref)	(ref)
Furloughed/laid off because store temp./permanently closed	1.69**	1.68**	0.27	0.29*
Furloughed/laid off for other reason	1.51***	1.42***	0.30**	0.31***
Demographic Controls	✔	✔	✔	✔
State Fixed-Effects		✔		✔
Employer Fixed-Effects	✔	✔	✔	✔
COVID-19 Exposure		✔		✔
Month Fixed-Effects		✔		✔
Round 9 (Fall, 2020) Data Only	✔	✔	✔	✔
(Mean)	(12.26)	(12.26)	(2.72)	(2.72)
N	4535	4535	4535	4535

Note: All models in Appendix Table C1 analyze data from Fall (2020) only. ***p < .001, **p < .01, *p < .05.

**Appendix Table C2:** Response to Reinterviews regressed on Respondent Attributes

	M1	M2	M3	M4
	UnweightedPredicted Response	UnweightedPredicted Response	WeightedPredicted Response	WeightedPredicted Response
	First Reinterview	Second Reinterview	First Reinterview	Second Reinterview
*Age*	0.00∗∗∗	0.00∗∗∗	0.00∗∗∗	0.00∗∗∗
*Race/Ethnicity*				
White, non-Hispanic	(ref)	(ref)	(ref)	(ref)
Black, non-Hispanic	−0.01	0.00	−0.02	−0.01
Hispanic	−0.02	−0.01	−0.00	0.01
Other race, non-Hispanic	−0.02	−0.00	−0.02	−0.00
*Gender*				
Female	0.01	0.01	0.02	0.02
*Partnership Status*				
Married, living with spouse	(ref)	(ref)	(ref)	(ref)
Living with a partner	−0.02	−0.02	−0.04∗	−0.03∗
Not living with a spouse or partner	−0.02	−0.00	−0.04∗	−0.01
*Child status*				
Has children	−0.04∗∗	−0.05∗∗∗	−0.05∗∗	−0.06∗∗∗
*Educational Attainment*				
Less than high school	(ref)	(ref)	(ref)	(ref)
High school diploma/GED	0.01	0.00	0.02	0.01
Some college	0.05∗	0.04∗	0.06∗	0.04
Associate's degree	0.10∗∗∗	0.08∗∗∗	0.09∗	0.08∗∗
Bachelor's degree	0.16∗∗∗	0.15∗∗∗	0.17∗∗∗	0.15∗∗∗
Master's or advanced degree	0.20∗∗∗	0.20∗∗∗	0.20∗∗∗	0.17∗∗∗
*School Enrollment*				
Enrolled in school	0.04∗∗	0.02∗	0.05∗∗	0.02
*English as a second language*	−0.01	−0.02	−0.02	−0.03
Observations	10,711	10,711	10,711	10,711

Note: All models In Appendix Table C2 analyze panel data. ∗∗∗p < .001, ∗∗p < .01, ∗p<0.05.

**Appendix Table C3:** Panel association between employment status and health outcomes.

	Sleep Quality				Happiness			
	1 = Very Good to 4 = Poor				1 = Very Happy to 3 = Not Too Happy			
	M1	M2	M3	M4	M1	M2	M3	M4
Not Employed	0.21**	0.16**	0.21**	0.15*	0.18***	0.12**	0.18***	0.13**
(Mean)	(2.85)	(2.85)	(2.85)	(2.85)	(2.21)	(2.21)	(2.21)	(2.21)
Demographic Controls at Baseline	✔	✔	✔	✔	✔	✔	✔	✔
Health at Baseline		✔		✔		✔		✔
Industry at Baseline			✔	✔			✔	✔
Panel Data	✔	✔	✔	✔	✔	✔	✔	✔
Observations	3307	3307	3307	3307	3307	3307	3307	3307

	Psychological Distress				Self-Rated Health			
	0 = None of the time to 20 = All of the time				1 = Excellent health to 5 = Poor health			
	M1	M2	M3	M4	M1	M2	M3	M4
Not Employed	2.35***	1.26**	2.37***	1.26**	0.30***	0.14*	0.28**	0.13*
(Mean)	(10.59)	(10.59)	(10.59)	(10.59)	(2.83)	(2.83)	(2.83)	(2.83)
Demographic Controls at Baseline	✔	✔	✔	✔	✔	✔	✔	✔
Health at Baseline		✔		✔		✔		✔
Industry at Baseline			✔	✔			✔	✔
Panel Data	✔	✔	✔	✔	✔	✔	✔	✔
Observations	3307	3307	3307	3307	3307	3307	3307	3307

Note: All models in Appendix Table C3 analyze panel data. ***p < .001, **p < .01, *p < .05.

**Appendix Table D1:** Association between unemployment insurance benefits and economic hardship/wellbeing by time period.

	M1	M2	M3	M4	M4	M4
	Hunger Hardship in Last Month	Utility Hardship in Last Month	Defer Medical Expenses	Housing Hardship	Difficulty Making Ends Meet	Lack Ability to Cope with $400 Shock
Employed	−0.17***	−0.19***	−0.23***	−0.15***	−0.43***	−0.37***
Not Employed, UI	−0.04	−0.05	−0.15**	−0.08**	−0.21***	−0.22**
Not Employed, No UI	(ref)	(ref)	(ref)	(ref)	(ref)	(ref)
Spring	−0.03	−0.08	−0.15***	−0.08**	−0.17**	−0.13*
Employed x Spring	−0.02	0.02	0.12*	0.06*	0.12*	0.10
Not Employed, UI x Spring	−0.14**	−0.07	0.05	0.01	−0.00	0.00
Not Employed, no UI x Fall	(ref)	(ref)	(ref)	(ref)	(ref)	(ref)
Demographic Controls	✔	✔	✔	✔	✔	✔
State Fixed-Effects	✔	✔	✔	✔	✔	✔
Employer Fixed-Effects	✔	✔	✔	✔	✔	✔
Pooled Data	✔	✔	✔	✔	✔	✔
(Mean)	(0.13)	(0.17)	(0.11)	(0.04)	(0.18)	(0.35)
N	12,483	12,483	12,483	12,483	12,483	12,483

Note: All models in Table D1 analyze pooled data. This includes Spring 2020 and Fall 2020. ***p < .001, **p < .01, *p < .05.

## Appendix C Analyses of Effect of Unemployment on Health and Wellbeing

We subject our estimates of the effect of job loss on worker health and wellbeing, presented in Table 1, to two further tests designed to provide additional causal leverage.

### *Store Closure and Wellbeing*

First, using information on the reasons for job loss, we estimate OLS models that contrast the wellbeing of those who remained employed with those who were furloughed/laid off due to store closure and to those who were furloughed/laid off for other reasons, again with employer fixed effects. If the apparent effect of job loss (from the models reported in Table 1) is spurious, we would expect to find little evidence of negative associations for those who lost their jobs due to establishment closure. But, if job loss has a true exogenous effect on wellbeing, we would expect to find robust negative associations for those who lost their job due to establishment closure.

To estimate these models, we draw on data collected at round 9 (Fall, 2020). Respondents were asked, “In [SURVEY-MONTH-YEAR], you told us you were working for [EMPLOYER-NAME]. Are you still working for [EMPLOYER-NAME]?”. Then, respondents who reported being furloughed or laid off were asked why that separation had occurred, with response options of “My workplace stayed open, but business was down due to the COVID-19 pandemic”, “My workplace closed temporarily due to the COVID-19 pandemic”, “My workplace closed permanently due to the COVID-19 pandemic”, “Temporary job that ended”, and “Other”. We construct a three-category variable that contrasts those employed with those who were furloughed or laid off because of either temporary or permanent store closure and with those who were furloughed or laid off for other reasons. Following the plant closure literature (Kessler et al., 1987; Schaller and Stevens 2014; Strully 2009), we expect store closure to be exogenous to individual characteristics that might be selective of job loss and poor health.

We estimate models that follow the same form as those reported in Table 1, including the full-set of respondent controls and the employer fixed-effects. We also estimate a version of the model that includes the control for COVID-19 exposure.

As shown in Appendix Table C1, we find that there are consistent negative effects of job loss due to establishment closure on happiness, psychological distress, and self-rated health. The coefficients reflecting the association between employment status and sleep quality are correctly signed and are of the same magnitude as those in M1-M4 of Table 1, but with the smaller sample size in Fall of 2020, are not statistically significant. Notably, these results also provide little evidence of selection into job loss for reasons other than establishment closure – the estimated coefficients are similar for both types of job loss.

### *Panel Estimates of the Effect of Job Loss on Health and Wellbeing*

Second, we draw on panel data collected from an occupational cohort of service sector workers who were employed at 106 large service sector firms in 2019 and who we recontacted between July and November of 2020 to produce longitudinal data on 3307 respondents.

To construct this cohort, we attempted to recontact 11,472 hourly service sector workers employed at 106 large firms (listed in Appendix Table A1) who were first surveyed by The Shift Project in rounds 6 and 7, that is in Spring or Fall of 2019, prior to the outbreak of the COVID-19 pandemic.

In July through November of 2020, we re-contacted these respondents using text message and email invitations to participate in a follow-up survey. Respondents were offered an escalating set of incentives to participation, beginning with entry into a drawing for a $500 gift card and escalating to a $25 payment. Of the 11,472 contacted, 3307 respondents participated in the reinterview.

To gauge the potential for bias in panel retention, we modeled panel retention as a function of a set of baseline characteristics. As shown in Appendix Table C2, we find those with children were less likely to respond to reinterview compared to those with no children. Those in school were more likely to respond than those not in school, and those with more educational attainment were more likely to respond. Race, gender, and cohabitation status were not predictive of retention. We adjust for all of these covariates in our models using the panel data.

The panel data provide two valuable benefits for analysis. First, while it is difficult to specify the selection process of those not employed into the cross-sectional surveys, the panel is based on an occupational cohort. Second, the repeated measures of health and wellbeing improve our capacity for causal inference. We use the panel data to estimate four models for each of the four outcomes, first with respondent controls, then introducing a lagged dependent variable (measured at baseline), then introducing industry fixed-effects, and then introducing both the lagged dependent variable and the industry fixed-effects.

As shown in Appendix Table C3, we find a consistent pattern of results across all outcomes, with the estimated effects of job loss smaller in the lagged dependent variable models, but still correctly signed and statistically significant.

## Appendix D Unemployment Insurance and Economic Outcomes

We expect that the degree to which not employed workers were buffered economically depends both on their success in accessing UI and on whether they did so during the period of augmented benefits in the Spring of 2020 or in the period of reversion in the Fall of 2020.

We show the implication of this heterogeneity in access to generous UI for household financial security in Economic Appendix Figures D1 and D2 by plotting estimates of household hardships in the prior month, difficulty making ends meet, and ability to cope with a $400 expense shock as a function of whether the survey occurred in the Spring (green line) (during the period of UI generosity) or in the Fall (orange line) of 2020 (after the expiration) as well as a function of whether the respondent was employed, not employed but did not receive UI, or not employed and received UI. These estimates are adjusted for the full set of respondent controls as well as employer and state fixed-effects. The detailed model results are presented in Economic Appendix Table D1.

In both Spring of 2020 and Fall of 2020, employed workers were the least likely to report going hungry at least once in the last month because they could not afford enough to eat – about 10% in each case – and not employed workers who had not received UI were the most likely to report such hunger hardship – about 30% in each case. But, there was a substantial difference in the rates of hunger hardship among those who received UI between the Spring and the Fall. In the spring, UI receipt fully buffered the effects of job loss on hunger, reducing hardship levels to the same as those of the employed. But, by Fall, when the augmented UI benefits had lapsed, this was much less the case, with UI receipt only somewhat reducing the effects of job loss on hardship. We see the same pattern play out for medical hardship, housing hardship, and, to a somewhat lesser extent, utility hardship. In the Spring of 2020, not employed respondents who received UI were no worse off than those who remained employed, while those who did not receive UI experienced significantly higher levels of hardship. But, in the Fall of 2020, the less generous UI benefits played a substantially smaller buffering role. The plots in Fig. 5 of difficulty making ends meet and of ability to cope with a $400 expense shock show the same pattern. In sum, effective access to UI in the Spring of 2020 fully buffered the negative economic effects of job loss. However, respondents who received the less generous UI benefits in the Fall were not fully buffered and respondents who applied for UI but had not heard back were much worse off economically in both the fall and the spring.

The results above show that workers who succeeded in receiving UI in the Spring of 2020 were no worse off economically than those who remained employed.
